# Exosomes from Short-Term High-Fat or High-Sucrose Fed Mice Induce Hepatic Steatosis through Different Pathways

**DOI:** 10.3390/cells12010169

**Published:** 2022-12-31

**Authors:** Carlos Castaño, Anna Novials, Marcelina Párrizas

**Affiliations:** 1 Pathogenesis and Prevention of Diabetes Group, Instituto de Investigaciones Biomédicas August Pi i Sunyer (IDIBAPS), 08036 Barcelona, Spain; 2 Pathogenesis and Prevention of Diabetes Group, Centro de Investigación Biomédica en Red de Diabetes y Enfermedades Metabólicas (CIBERDEM), 08036 Barcelona, Spain

**Keywords:** exosome, miRNA, obesity, MAFLD, biomarker

## Abstract

Obesity and other closely associated diseases, such as metabolic-associated fatty liver disease (MAFLD) and type 2 diabetes, give rise to a common biometric and metabolic phenotype resulting from a different etiopathogenesis. To characterize the first stages of metabolic dysfunction induced by either obesity or hepatic steatosis, we compared two animal models of short-term feeding with either high-fat (HFD) or high-sucrose (SAC) diets. Using transcriptomic, metabolic, and calorimetric analyses, we determined that a short-term HFD leads to obesity and then hepatic steatosis through lipid storage, whereas SAC increases gluconeogenesis and de novo lipogenesis, resulting in hepatic steatosis followed later by obesity. Plasma exosomal miRNA profiles differed between HFD and SAC mice, and the injection of exosomes from HFD or SAC mice reproduced some transcriptomic and metabolic features of the donor mice. Finally, we exploited our data to identify circulating *miR-22-3p* as a candidate biomarker for MAFLD patient stratification. In conclusion, dietary challenges affecting adipose or hepatic metabolism regulate the abundance of exosomal miRNAs in plasma, which in turn modulate gene expression, helping the organism to adapt.

## 1. Introduction

Type 2 diabetes (T2D) is a highly prevalent, multifactorial disorder with a complex etiology that debuts when pancreatic β-cells fail to produce enough insulin to compensate for insulin resistance [1]. Defects of insulin action in different tissues or alterations in β-cell secretory capacity may underlie the development of the disease in different individuals. Extensive phenotyping of patients with T2D has consistently identified abdominal obesity, hepatic steatosis, or the genetic alterations associated with deficient β-cell function as the key triggering defects [2,3,4]. Obesity, described as excess fat mass accumulation, is closely associated with the development of cardiometabolic disorders, including T2D and metabolic-associated fatty liver disease (MAFLD) [5]. Moreover, MAFLD and T2D are intimately connected [6]. A calorie-dense diet, such as the Western diet, coupled with low activity rates causes obesity [7], which is often associated with an unhealthy expansion of adipose tissue, leading to chronic subclinical inflammation, anomalous adipokine secretion, and ultimately hepatic steatosis [8]. On the other hand, the increased consumption of sweetened beverages and processed foods with an elevated high-fructose corn syrup content boosts hepatic de novo lipogenesis (DNL), leading to MAFLD and, frequently, obesity and T2D [9]. Hence, obesity and hepatic steatosis influence each other and may give rise to similar phenotypes of peripheral insulin resistance, leading to hyperglycemia and even triggering T2D. Being able to stratify patients according to their etiology is essential to personalizing treatment and achieving maximal response to therapy, while minimizing undesirable adverse effects [10]. However, this is made difficult by the interconnections among tissues. When the metabolic functionality of a tissue is disturbed, the effects of that alteration influence other tissues, ultimately leading to impaired global metabolic homeostasis and the original malfunction being masked [11].

Exosomes, small vesicles released by most cells and readily detected in biological fluids, transfer bioactive material, including miRNAs among cells. Exosomal miRNAs (exo-miRNAs) regulate the transcriptome of the cells that capture them, thus participating in inter-tissue crosstalk [12,13,14,15]. We, and others, have shown that the miRNA cargo of the exosomes circulating in blood, reflecting the contribution of many tissues, is altered by environmental challenges such as diet or exercise [13,14,16,17,18]. Here, we aimed to characterize the first stages of the metabolic dysfunction induced by either obesity or hepatic steatosis by comparing two animal models of short-term feeding with either a high-fat diet or sucrose-supplemented drinking water. We examined the role of exo-miRNAs in the establishment of the pathological phenotype and how this information may be translated into a new tool for patient stratification.

## 2. Materials and Methods

### 2.1. Animal Studies

Procedures were conducted in accordance with the principles of laboratory animal care (European and local government guidelines) and approved by the Animal Research Committee of the University of Barcelona (register number: 46/18). Metabolic tests and biochemical measurements were performed as described [13,14,16]. Fat distribution was analyzed using magnetic resonance imaging (MRI) using a BioSpec 70/30 scanner [19]. Gas exchange was measured using the CaloSys TSE Systems [14,16]. When indicated, mice were pretreated with an intraperitoneal (IP) injection of 10 mg/Kg C75 1 h before the tests [20]. Donors: C57BL/6J male mice were fed standard chow (CT), standard chow with drinking water supplemented with sucrose (SAC), or a high-fat diet (HFD, 45% fat) for 6 or 15 weeks. Four independent cohorts (n = 6/group each) were analyzed at the 6-week point and two more at the 15-week point (n = 4/group). Injected: C57BL/6J control male mice were injected intravenously (IV) with exosomes isolated from the plasma of CT (iCT), SAC (iSAC), or HFD (iHFD) mice. The first cohort received a single injection (acute treatment, n = 4/group), whereas the second cohort received 3 injections separated by 4 days (chronic treatment, n = 4/group). Biodistribution: C57BL/6J male mice overexpressing enhanced green fluorescent protein (EGFP) (Jackson) [21] were injected IV with exosomes transfected with 500 pmol siEGFP [13,14,16], or with siEGFP coupled with Invivofectamine (n = 3/group). The animal experiment scheme is presented in Supplementary Figure S1A.

### 2.2. Exosome Characterization

Exosomes were isolated from 500 μL of plasma [13,14,16]. Interstitial liver exosomes were isolated as described [14,22]. Prior to ultracentrifugation, liver supernatants were cleaned using SmartSEC columns. Exosomes were characterized by Bradford assay, acetylcholinesterase ELISA, nanoparticle tracking analysis (NTA), transmission electron microscopy (TEM), and Western blotting [13,14,16].

### 2.3. Protein Analyses

Fixed tissues were embedded in optimal cutting temperature (OCT) compound (liver) or paraffin (adipose). Stainings were performed as described [13,14,16]. For the biodistribution studies, liver and gastrocnemius were embedded in OCT to obtain 10 µm sections, whereas adipose was minced and applied to a slide. For Western blotting, protein was extracted with RIPA, resolved in 7.5% precast protein gels, and transferred to nitrocellulose membranes. Chemiluminescence was detected with an enhanced chemiluminescence (ECL) substrate in an LAS4000 Lumi-Imager.

### 2.4. RNA Analyses

Tissue RNA was extracted with an miRNeasy Mini Kit and analyzed using hybridization on Clariom™ S HT mouse array plates. Microarray data was analyzed with Transcriptome Analysis Console Software. Exosomal RNA was extracted from 25 µL plasma or liver exosomes with miRNeasy and miRNA profiling performed by real time RT-PCR using mouse/rat miRNome plates. Differential expression was determined with GenEx.

### 2.5. Clinical cohort

Serum samples were obtained from the Hospital Clínic-IDIBAPS Biobank. Participants gave written informed consent, and studies were approved by the Research and Ethics Committees of the Hospital Clinic, University of Barcelona (register 2011/6945). Circulating miRNAs were isolated and analyzed as published in [23]. For the current study, the subjects (n = 40) were distributed according to the fatty liver index (FLI), calculated as described [23,24]. Receiver operating characteristic (ROC) curves were plotted using ROC Station, and the area under the curve (AUC) was calculated to determine discrimination accuracy.

### 2.6. Statistics

The differences between groups were determined by either *t*-test analysis, when only two groups were compared, or by one-way ANOVA with *t*-test analysis, for pairwise comparison of three or more groups with a different number of values. The Shapiro–Wilk test *p*-value was calculated to ascertain the normality of the samples.

## 3. Results

### 3.1. High-Fat and High-Sucrose Diets Lead to a Convergent Obese Phenotype over Time

**Figure 1:**
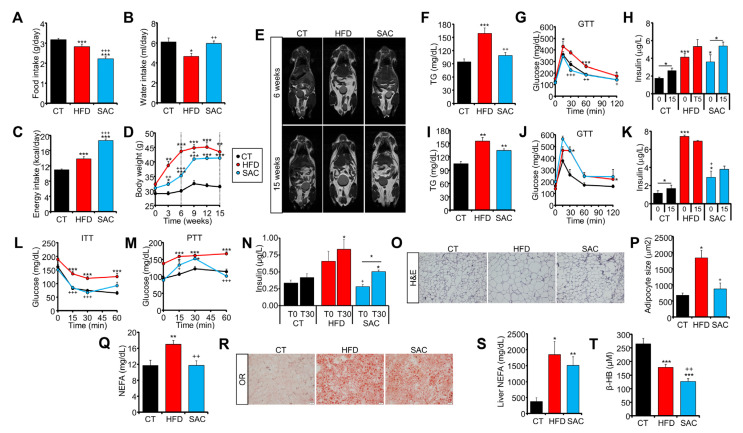
High-fat and high-sucrose diets lead to a convergent obese phenotype over time. (**A**–**C**): Food (**A**) and drink (**B**) intake and calories consumed (**C**) by male C57BL/6J mice fed either standard chow (CT), a high-fat diet (HFD), or standard chow with drinking water supplemented with 50% sucrose (SAC). (**D**) Body weight of the same mice during 15 weeks of diet. (**E**) Magnetic resonance imaging (MRI) analysis of fat distribution of the same mice after 6 (top panels) and 15 weeks (lower panels) of diet. (**F**–**H**): After 6 weeks, HFD mice are hypertriglyceridemic (**F**), glucose intolerant (**G**), and hyperinsulinemic (**H**), whereas SAC mice are hyperinsulinemic. (**I**–**K**): After 15 weeks, both HFD and SAC mice are hypertriglyceridemic (**I**), glucose intolerant (**J**), and hyperinsulinemic (**K**). (**L**) After 6 weeks, HFD mice, but not SAC mice, are insulin resistant according to an insulin tolerance test (ITT). (**M**,**N**): SAC mice show increased gluconeogenesis (**M**) and insulin release (**N**) during a pyruvate tolerance test (PTT), evidencing hepatic insulin resistance. (**O**–**Q**): Epididymal white adipose tissue (eWAT) of HFD mice shows adipocyte hypertrophy (**O**,**P**) and increased levels of plasma non-esterified fatty acids (NEFA) (**Q**). (**R**–**T**): Livers of both HFD and SAC mice show hepatic steatosis (**R**), increased NEFA content (**S**) and decreased levels of β-hydroxybutyrate (β-HB) (**T**). n = 6/group (**A**–**D**,**F**–**N**,**Q**,**S**,**T**), n = 3/group (E), n = 4/group (**O**,**P**,**R**). * *p* < 0.05, ** *p* < 0.01, and *** *p* < 0.005 with respect to the CT group unless otherwise indicated. + *p* < 0.05, ++ *p* < 0.01, and +++ *p* < 0.005 with respect to the HFD group. GTT = glucose tolerance test; H&E = hematoxylin and eosin; OR = oil red; TG = triglycerides.

To separate the effects of obesity and hepatic steatosis on metabolism, we fed C57BL/6J male mice with either a high-fat diet (HFD, 45% fat) or with standard chow and drinking water supplemented with 50% sucrose (SAC). Mice fed with standard chow and water were used as the control (CT). HFD mice ate less solid food and drank less water than CT mice (Figure 1A,B). However, due to the higher density of the food, the calories ingested were higher, and HFD mice rapidly gained more weight than the other groups (Figure 1C,D). In contrast, SAC mice ate less solid food than the other groups but drank the same amount of water as CT mice (Figure 1A,B), resulting in a higher caloric intake due to sucrose ingestion (Figure 1C). Interestingly, SAC mice gained weight more slowly than HFD mice, but by week 15, both groups were indistinguishable (Figure 1D). The visualization of fat distribution by MRI confirmed that HFD mice had larger fat depots after 6 weeks of the diet (Figure 1E, Supplementary Figure S1B), but after 15 weeks, HFD and SAC mice showed similar adiposity (Figure 1E, Supplementary Figure S1C). Moreover, after 6 weeks, HFD mice had higher blood triglycerides (TG) and impaired glucose tolerance, even though they were hyperinsulinemic (Figure 1F–H). Although, at this point, SAC mice had normal glucose tolerance, they were also hyperinsulinemic, suggesting insulin resistance (Figure 1G,H), which was further supported by similar homeostatic model assessment-insulin resistance (HOMA-IR) levels as HFD mice (Supplementary Figure S1D). Surprisingly, most of the differences between HFD and SAC mice were lost after 15 weeks, with both groups then showing increased blood TG and glucose intolerance (Figure 1I,J), although HFD mice were even more hyperinsulinemic (Figure 1K).

We then focused on HFD and SAC mice after 6 weeks of diet, when they were phenotypically different. HFD mice were insulin resistant according to an insulin tolerance test (ITT), whereas SAC mice displayed normal insulin tolerance (Figure 1L). However, as mentioned, their increased insulin levels point to insulin resistance (Figure 1H, Supplementary Figure S1D), and a pyruvate tolerance test (PTT) showed higher substrate-induced hepatic glucose production in SAC mice than in CT mice (Figure 1M), even in the presence of significantly higher levels of insulin during the test (Figure 1N). These data suggest hepatic insulin resistance in SAC mice, whereas HFD mice, which maintained high glucose levels during fasting and throughout the test, show more extensive peripheral insulin resistance.

Tissue analysis shows that liver weight was significantly increased in SAC mice after 6 weeks, whereas the epididymal (eWAT) and subcutaneous (sWAT) white adipose tissues were enlarged in HFD mice (Supplementary Figure S1E). Even though both HFD and SAC mice had significantly increased body weight compared with the CT group at this time point (Figure 1D), histological analysis of eWAT evidenced adipocyte hypertrophy only in HFD mice (Figure 1O,P, Supplementary Figure S1F), in accordance with a higher degree of obesity in this group than in SAC mice. Moreover, HFD mice also had increased circulating non-esterified fatty acids (NEFA) (Figure 1Q). On the other hand, both HFD and SAC groups had hepatic steatosis (Figure 1R,S, Supplementary Figure S1G), high plasma alanine transaminase (ALT) activity (Supplementary Figure S1H), and low plasma β-hydroxybutyrate (β-HB), a proxy for liver β-oxidation (Figure 1T). Remarkably, glucose tolerance, as measured by the AUC during the glucose tolerance test (GTT), significantly correlated with body weight across all groups and time points (Supplementary Figure S1I), suggesting that adipose enlargement is more closely linked to global glucose tolerance than hepatic steatosis.

Hence, administrations of either high-fat or high-sucrose diets are both associated with similar metabolic disturbances in the long term, while presenting specific features early on. We focused on the effects after 6 weeks of diet, when HFD mice were already frankly obese, whereas SAC mice, although heavier than CT mice, had normal adipose histological features, to analyze how alterations in adipose or liver function contribute to the development of metabolic disease.

### 3.2. HFD and SAC Induce Distinct Gene Expression Profiles in eWAT and Liver

**Figure 2:**
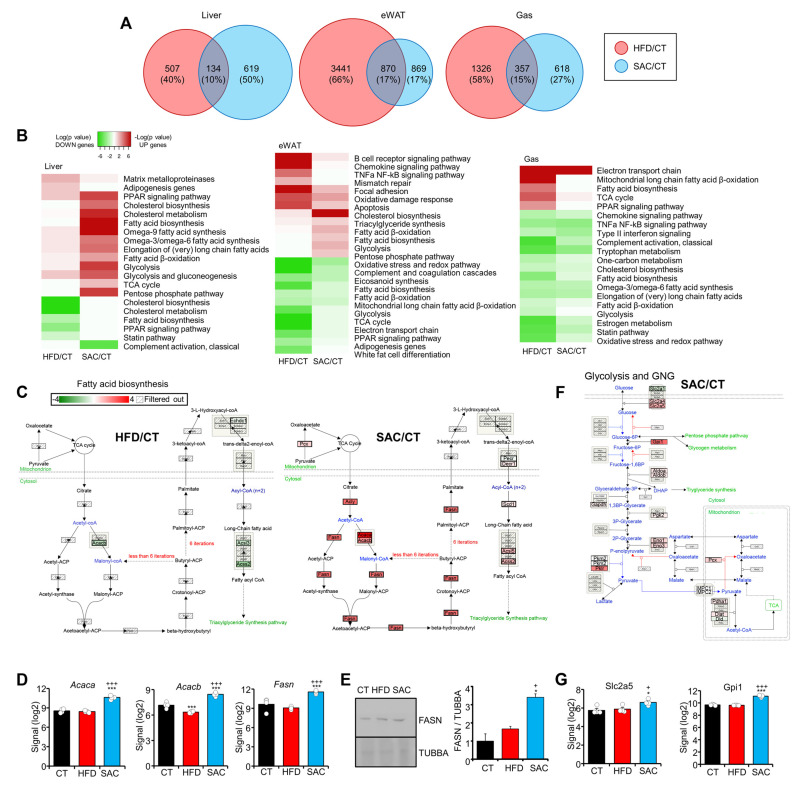
HFD and SAC induce distinct gene expression profiles in eWAT and liver. (**A**) Comparison of DEGs in HFD/CT and SAC/CT for each tissue analyzed using microarray hybridization. (**B**) Enrichment analysis showing the log (*p* value) of pathways associated with upregulated (red) or downregulated genes (green) in the same tissues of HFD and SAC mice. (**C**–**E**): The fatty acid biosynthesis pathway is associated with downregulated genes in HFD mice and with upregulated genes in SAC mice (**C**), including FASN, as determined by mRNA (**D**) and protein levels (**E**). (**F**,**G**): The glycolysis and gluconeogenesis (GNG) pathway is associated with upregulated genes only in SAC mice (**F**) including fructose handling genes such as *Slc2a5* (**G**). n = 4/group (**A**–**D**,**G**) and n = 3/group (**E**). * *p* < 0.05, and *** *p* < 0.005 with respect to the CT group. + *p* < 0.05, and +++ *p* < 0.005 with respect to the HFD group. Gas = gastrocnemius.

We analyzed the global gene expression in the liver, eWAT, and gastrocnemius (Gas) of CT, HFD, and SAC mice (n = 4/group) using microarray hybridization. Remarkably, eWAT showed a higher number of differentially expressed genes (DEGs) in both models; however, liver was the tissue most different, with only 10% common DEGs (Figure 2A, Supplementary Figure S2A). Principal component analysis (PCA) and the hierarchical clustering of DEGs clearly differentiated the SAC group from the other two in the case of liver, whereas HFD mice were separated in the case of eWAT and Gas (Supplementary Figure S2B,C).

Pathway analysis of the DEGs highlights the enrichment of lipid metabolic pathways among the upregulated genes in the liver of SAC mice (Figure 2B, Supplementary Table S1). Fatty acid biosynthesis, including the key enzymes *Acaca*, *Acacb*, and *Fasn*, was remarkably increased (Figure 2C–E, Supplementary Figure S2D), as was cholesterol biosynthesis (Supplementary Figure S2E,F). Interestingly, both pathways were downregulated in HFD mice, evidencing that, even though both groups displayed hepatic steatosis, the pathways leading to it were different. Moreover, the glucose metabolic pathways, including glycolysis and gluconeogenesis (GNG), the tricarboxylic acid cycle, and the pentose phosphate pathway, were also upregulated, specifically in the liver of SAC mice (Figure 2B,F, Supplementary Figure S2G). Remarkably, the genes involved in fructose utilization, including transporter *Slc2a5* and enzyme *Gpi1*, were increased only in the liver of SAC mice (Figure 2G).

On the other hand, adipose inflammation was evident only in HFD mice (Figure 2B, Supplementary Figure S2H,I, Table S2), in accordance with tissue enlargement and adipocyte hypertrophy (Figure 1E). Additionally, the Gas in HFD mice showed an increased fatty acid biosynthesis pathway (Figure 2B, Supplementary Table S3), which is often associated with lower insulin sensitivity and glucose tolerance.

Overall, these data suggest that the role of the liver is eminently different in the HFD and SAC models, even though both display hepatic steatosis.

### 3.3. SAC Mice Rely on DNL to Maintain Glucose Homeostasis

**Figure 3:**
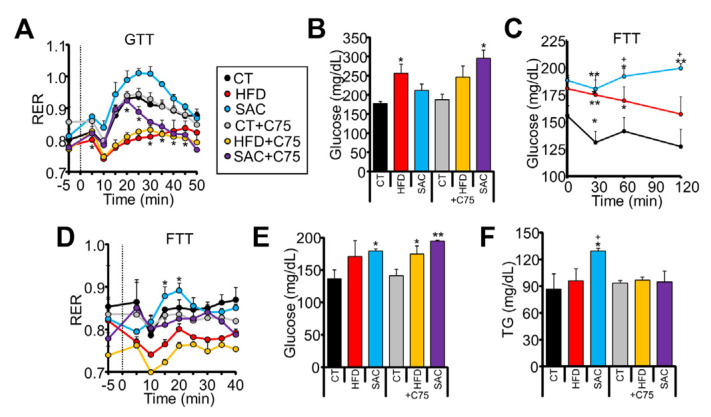
SAC mice rely on de novo lipogenesis (DNL) to maintain glucose homeostasis. (**A**) Oral administration of glucose increased respiratory exchange ratio (RER) in CT but not HFD mice, as measured by indirect calorimetry. SAC mice show significantly higher levels, which are reduced by pretreatment with FASN inhibitor C75. (**B**) At the end of the calorimetric test shown in (**A**), only HFD mice show high glucose levels, but after pretreatment with C75, SAC mice also show increased glycemia. (**C**,**D**): Oral administration of fructose increase glucose (**C**) and RER only in SAC mice (**D**), and the increased RER is blunted by pretreatment with C75. (**E**,**F**): At the end of the calorimetric test shown in (**D**), SAC mice show increased glycemia and triglyceridemia, whereas C75 pretreatment further increases glycemia (**E**) and decrease TG (**F**). n = 3/group (**A**,**B**,**D**,**F**), n = 6/group (**C**). * *p* < 0.05, and ** *p* < 0.01 with respect to the CT group. + *p* < 0.05 with respect to the HFD group. FTT = fructose tolerance test.

We performed indirect calorimetry assays to dissect how HFD and SAC mice handle ingested glucose. The mice were subjected to a GTT while gas exchange was measured on an airtight, stationary treadmill. Oral glucose administration increased the respiratory exchange ratio (RER) above 0.9 in CT mice, indicative of carbohydrate oxidation (CHO) (Figure 3A). HFD mice were unable to increase their RER, suggesting metabolic inflexibility. Surprisingly, SAC mice reached RER values near or above 1.0 (Figure 3A, Supplementary Figure S3A), proposed to be indicative of DNL [25]. Hence, we repeated the test while pre-treating the mice with FASN inhibitor C75. FASN inhibition did not affect the RER values of CT or HFD mice, but significantly decreased those of SAC mice (Figure 3A). Importantly, in the absence of the inhibitor, HFD mice, but not SAC mice, displayed glucose levels higher than CT mice, as expected from the GTT (Figure 1G); however, in the presence of C75, SAC mice were unable to control glycemia (Figure 3B), pointing to a role for DNL in maintaining glucose homeostasis in this group. Moreover, SAC mice showed higher energy expenditure (EE) than the other groups (Supplementary Figure S3B), which may help explain the slower weight gain dynamics of this diet compared with a HFD (Figure 1D) [26].

We then analyzed the response to fructose administration. It has been shown that mice adapted to a high-fructose diet have an enhanced capacity to transform fructose to glucose [27]. Accordingly, we observed that only SAC mice had increased plasma glucose levels after oral fructose administration (Figure 3C). Moreover, when monitored by indirect calorimetry, only SAC mice significantly increased their RER from their basal levels in response to fructose (Figure 3D). Treatment with C75 again blunted RER increase (Figure 3D) and resulted in even higher glycemia (Figure 3E), whereas plasma TG levels, which were increased by fructose only in the SAC group, failed to increase (Figure 3F). These data suggest that SAC mice are adapted to increase DNL in response to sucrose ingestion, and this pathway is needed to maintain glucose homeostasis.

### 3.4. Injection of HFD and SAC Plasma Exosomes Reproduce Transcriptomic Changes Observed in Donor Mice

**Figure 4:**
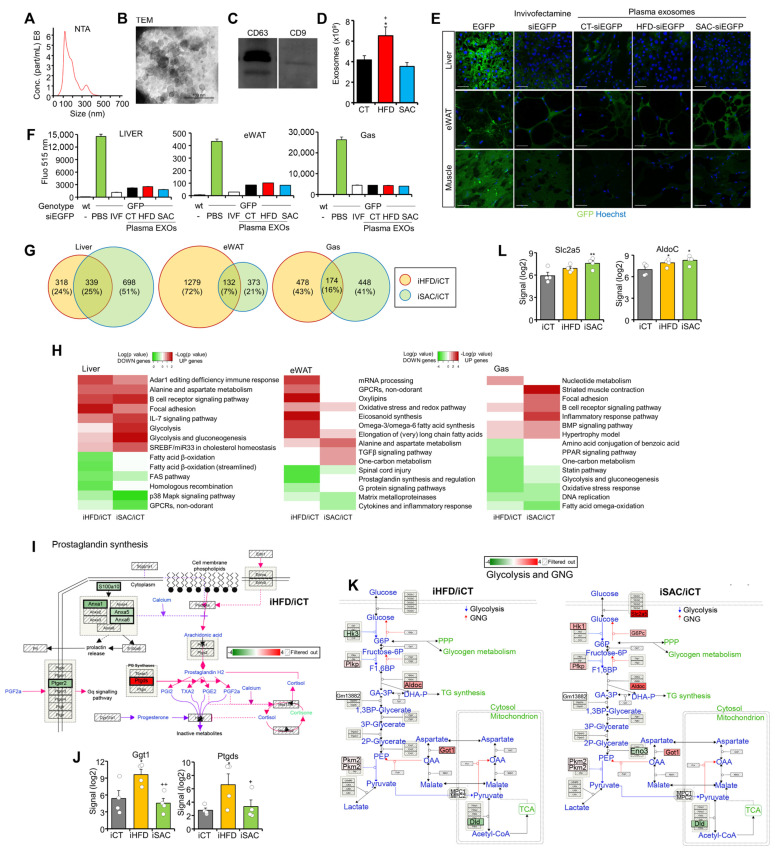
Injection of HFD and SAC plasma exosomes reproduce transcriptomic changes observed in donor mice. (**A**–**D**): Plasma exosomes from donor mice were characterized by NTA (**A**), TEM (**B**), Western blotting of membrane markers CD63 and CD9 (**C**), and exosome number (**D**). (**E**,**F**): Biodistribution of plasma exosomes from CT, HFD, and SAC mice was followed by transfecting them with a siRNA against EGFP and IV injection into EGFP transgenic mice. Injection of the same siEGFP associated with Invivofectamine was used as a positive control. Loss of fluorescence was determined using confocal microscopy of the tissues of interest (**E**) and luminometer analysis of tissue lysates (**F**). (**G**) Comparison of DEGs in mice injected with HFD plasma exosomes (iHFD) or SAC exosomes (iSAC) with those injected with CT exosomes (iCT) for each tissue analyzed using microarray hybridization. (**H**) Enrichment analysis showing log (*p* value) of pathways associated with upregulated (red) or downregulated genes (green) in the same tissues of iHFD and iSAC mice. (**I**,**J**): Inflammation is increased in the eWAT of iHFD mice, with decreased prostaglandin synthesis (**I**) and increased eicosanoid synthesis genes (**J**). (**K**,**L**): The glycolysis and GNG pathway is increased in the liver of iSAC mice (**K**), particularly those genes involved in fructose handling (**L**). n = 3/group (**A**–**F**) and n = 4/group (**G**–**L**). * *p* < 0.05, and ** *p* < 0.01 with respect to the iCT group. + *p* < 0.05, and ++ *p* < 0.01 with respect to the iHFD group. Scale bar in (**E**) represents 50 µm. EGFP = enhanced green fluorescent protein; IVF = invivofectamine; NTA = nanoparticle tracking analysis; TEM = transmission electron microscopy.

We have demonstrated that exo-miRNAs participate in the regulation of metabolic homeostasis [13,14,16]. Hence, we isolated exosomes from CT, HFD, and SAC mice plasma and characterized them using NTA, TEM, Western blotting of classical exosome markers CD63 and CD9, acetylcholinesterase activity, and protein concentration (Figure 4A–D, Supplementary Figure S4A). We then performed a biodistribution study to ascertain that the nucleic acid content of exogenously administered exosomes is not only able to reach the tissues of interest, but can also exert a measurable phenotypical effect. We injected transgenic mice overexpressing EGFP in all tissues except erythrocytes and hair [21] with 50 µg exosomes from CT, HFD, or SAC mice transfected with 500 pmols siRNA targeting EGFP (siEGFP). As a control, we treated the mice with the same siEGFP coupled with Invivofectamine. The mice were sacrificed 72 h afterwards and tissue fluorescence was analyzed (Figure 4E,F, Supplementary Figure S4B). Confocal analysis demonstrated that the exosomes had a broad biodistribution, with decreased fluorescence observed in the liver, eWAT, and gastrocnemius (Figure 4E). Fluorescence quantification of equal quantities of tissue lysate also showed a significant decrease in the exosome-treated mice. Exosomes induced a similar effect to Invivofectamine, and no differences were observed among the exosomes prepared from the different diet groups (Figure 4F).

As exosome protein concentration strongly correlated with exosome number (Supplementary Figure S4A), we injected the control mice fed standard chow IV with 200 µg plasma exosomes, corresponding to approximately 10^11^ vesicles, from CT, HFD, or SAC mice to study their role in the development of the metabolic phenotypes observed in the donor mice. The injected mice were sacrificed 72 h after the injection, and gene expression was analyzed in the liver, eWAT and Gas using microarray hybridization. Remarkably, the mice injected with SAC exosomes (iSAC) displayed the greatest transcriptomic changes in liver, whereas eWAT was the most affected tissue in the mice injected with HFD exosomes (iHFD) (Figure 4G, Supplementary Figure S4C). PCA was unable to distinguish among groups (Supplementary Figure S4D), but the hierarchical clustering of DEGs separated iCT livers and Gas from the two experimental groups, while the iHFD eWAT tended to separate from the other two groups (Supplementary Figure S4E).

Enrichment analysis (Supplementary Tables S4–S6) identified increased lipid metabolism in iHFD eWAT (Figure 4H), with decreased prostaglandin synthesis (Figure 4I, Supplementary Figure S4F) and increased eicosanoid synthesis (Figure 4J, Supplementary Figure S4G). However, the most surprising result was the increased glycolysis and GNG pathway in the liver of iSAC mice (Figure 4H,K), with an upregulation of the genes involved in fructose handling, such as transporter *Slc2a5* and enzyme *AldoC* (Figure 4L). Hence, exosomes from the short-term HFD and SAC models transmitted some of the transcriptional changes of the donor mice when transferred to control mice.

### 3.5. Injection of HFD and SAC Plasma Exosomes Reproduce the Metabolic Phenotype Observed in Donor Mice

**Figure 5:**
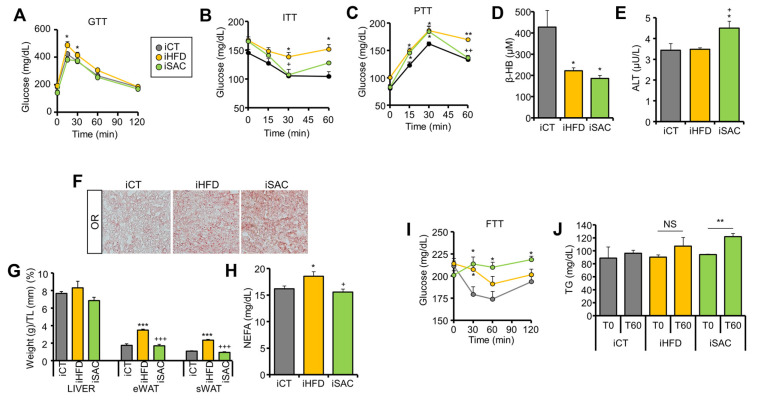
Injection of HFD and SAC plasma exosomes reproduce the metabolic phenotype observed in donor mice. (**A**,**B**): iHFD but not iSAC show glucose intolerance (**A**) and insulin resistance (**B**). (**C**,**D**): Both experimental groups show increase glucose output during a PTT (**C**), and lower circulating β-HB levels (**D**). (**E**) Only iSAC mice have increased alanine transaminase (ALT) activity. (**F**) Both experimental groups display hepatic steatosis. (**G**,**H**): Only iHFD mice show significant enlargement of adipose depots (**G**) and elevated circulating NEFA levels (**H**). (**I**,**J**): Oral administration of fructose increased glycemia (**I**) and circulating TG (**J**) only in iSAC mice. n = 4/group (**A**–**J**). * *p* < 0.05, ** *p* < 0.01, and *** *p* < 0.005 with respect to the iCT group. + *p* < 0.05, ++ *p* < 0.01, and +++ *p* < 0.005 with respect to the iHFD group. NS = not significant.

To determine if the transcriptomic changes observed after exosome injection translate into phenotypical effects, we performed a prolonged treatment with the same exosome preparations. We treated control mice with 3 IV injections separated by 4 days, a first injection of 100 µg and two more of 50 µg each. Metabolic characterization was performed starting 15 days after the first injection. Interestingly, only iHFD mice presented a degree of glucose intolerance (Figure 5A) and significant insulin resistance (Figure 5B), with fasting hyperglycemia (Supplementary Figure S5A), as observed in the donor HFD mice. On the other hand, both iHFD and iSAC mice showed increased hepatic glucose production during a PTT (Figure 5C). Additionally, β-HB plasma levels were decreased in both models (Figure 5D), whereas only iSAC mice showed increased ALT activity (Figure 5E). Fasting TG levels remained unchanged (Supplementary Figure S5B), although both groups displayed hepatic steatosis (Figure 5F). Importantly, even though neither treatment affected body weight (Supplementary Figure S5C), iHFD mice showed enlarged eWAT and sWAT depots, and incremented circulating NEFAs (Figure 5G,H).

Notably, iSAC mice submitted to oral fructose administration were the only group able to transform fructose into glucose (Figure 5I), and this was further associated with increased plasma TG (Figure 5J), in accordance with the increased glycolysis and GNG pathway activation observed, particularly the elevated expression of fructose handling enzymes (Figure 4K,L).

Overall, exosomes from HFD mice primarily affected the adipose phenotype, as expected from the transcriptomic changes (Figure 4G), resulting in secondary hepatic steatosis due to increased circulating NEFA, as previously observed [13], whereas the primary target of SAC exosomes is the liver, favoring GNG and DNL, ultimately resulting in hepatic steatosis. All these data suggest that exosomes help adapt the metabolism to environmental challenges.

### 3.6. The miRNA Profile Differs between HFD and SAC Plasma and Liver Exosomes

**Figure 6:**
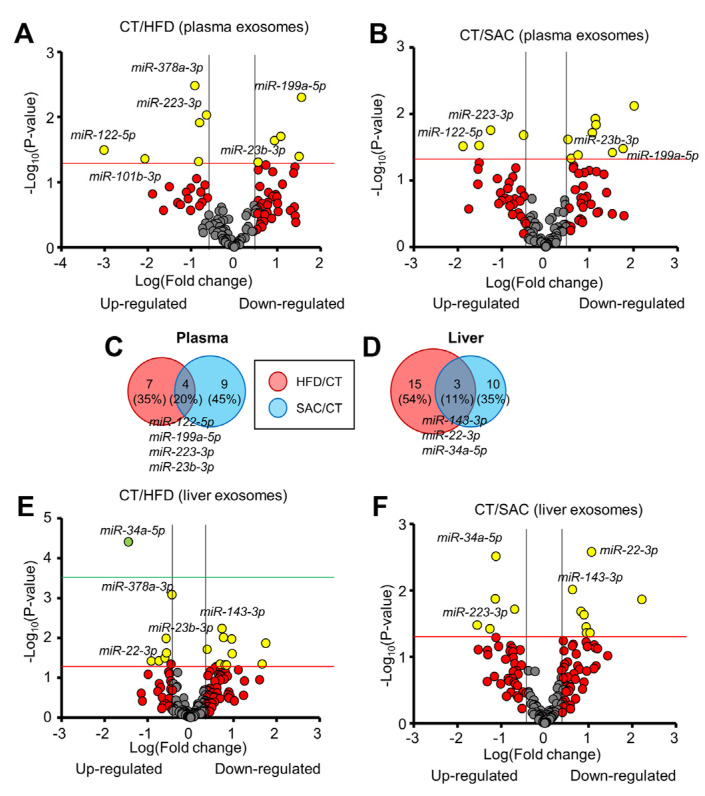
The miRNA profile differs between HFD and SAC plasma and liver exosomes. (**A**,**B**): Volcano plots show differential miRNA expression of plasma exosomes from HFD (**A**) and SAC mice (**B**) compared with CT mice. (**C**,**D**): Comparison of the differentially expressed exosomal miRNAs in HFD/CT and SAC/CT in plasma (**C**) and liver (**D**). (**E**,**F**): Volcano plots show differential miRNA expression of liver exosomes from HFD (**E**) and SAC mice (**F**). n = 3/group (**A**–**C**) and n = 4/group (**D**–**F**).

We proceeded to determine the plasma exo-miRNA profiles of HFD and SAC mice. A total of 123 miRNAs from the 375 measured by RT-PCR were detected. We identified 11 and 13 miRNAs whose abundance was significantly altered in HFD and SAC exosomes, respectively (Figure 6A,B, Supplementary Tables S7 and S8), with 4 miRNAs regulated in the same direction in both conditions: *miR-122-5p* and *miR-223-3p* upregulated and *miR-199a-5p* and *miR-23b-3p* downregulated (Figure 6C). Remarkably, we had previously described the upregulation of *miR-122-5p* in plasma exosomes from obese mice after 15 weeks of a HFD [13]. Indeed, all three more upregulated miRNAs in HFD mice after 6 weeks of diet remained upregulated after 15 weeks.

As liver was the tissue showing the most gene expression differences among donor HFD and SAC mice, we wanted to determine if hepatic exo-miRNAs were responsible for some of the changes in miRNA abundance observed in plasma exosomes. We isolated interstitial exosomes from liver (Supplementary Figure S6A) [14,22]. To ensure that the hepatic exosome samples were not contaminated with tissue debris or intracellular miRNAs, we cleaned them using size exclusion chromatography and RNAse treatment. RNA was analyzed using real-time RT-PCR from hepatic exosomes and liver tissue from CT mice. Most of the miRNAs detected in the tissue were also detected in the exosomes (Supplementary Figure S6B), but the miRNA profile of both types of samples greatly differed (Supplementary Figure S6C, Table S9). As expected, snRNAs (*U6*, *RN1A1*, and *RNU5G*) were highly enriched in tissue samples compared with the exosomes, thus indicating a low degree of contamination.

A total of 162 miRNAs were detected in at least one type of exosome sample in the CT group, with 73.5% common to plasma and liver exosomes (Supplementary Figure S6D). Interestingly, 4 miRNAs were detected only in plasma exosomes, whereas the 39 detected in hepatic exosomes were undetectable in plasma. By comparing the hepatic exo-miRNA profiles for HFD and SAC mice, only three miRNAs (11%) showed overlap, with *miR-34a-5p* upregulated, *miR-143-3p* downregulated, and, surprisingly, *miR-22-3p* showing opposite regulation in each condition (Figure 6D–F, Supplementary Tables S10 and S11).

A comparison of the regulated exo-miRNAs for each population and condition showed plasma/liver overlaps for *miR-378a-3p* among the upregulated and *miR-23b-3p* among the downregulated miRNAs in HFD mice. In the case of SAC mice, we only observed a common upregulation for *miR-223-3p* in liver and plasma exosomes (Supplementary Figure S6E).

These data indicate that the plasma exo-miRNA profile after 6 weeks of a HFD resembles the profile of frankly obese mice after 15 weeks. Moreover, the profile of SAC mice differs from that of HFD mice, despite sharing some metabolic features. Regarding hepatic exosomes, their miRNA profile is even more different between groups. Unfortunately, we were not able to detect a clear relationship between the regulation of hepatic and plasma exo-miRNAs.

### 3.7. Circulating miR-22-3p Can Be Used as a Biomarker for MAFLD Stratification

**Figure 7:**
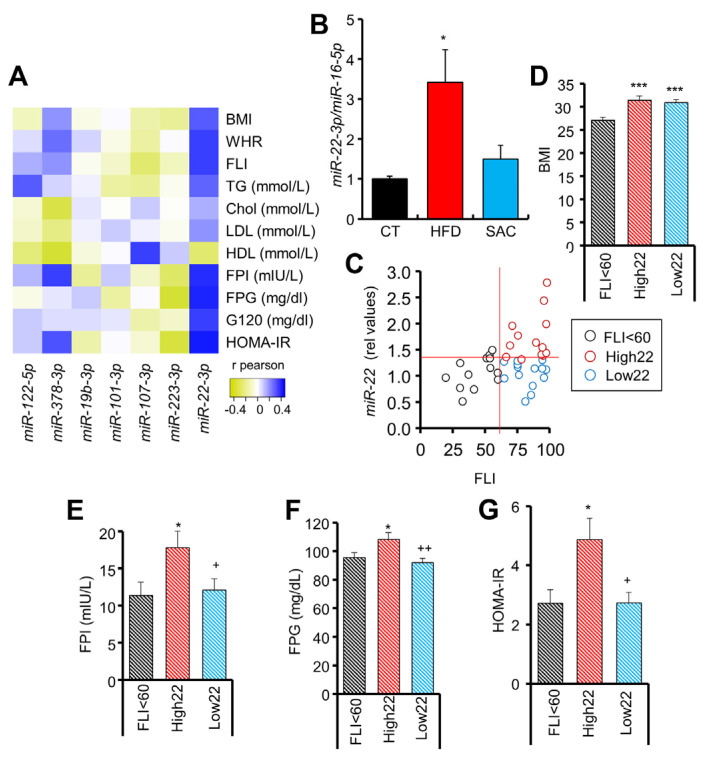
Circulating *miR-22-3p* can be used as a biomarker for MAFLD stratification. (**A**) Heatmap showing positive (blue) or negative (yellow) correlation coefficient values between circulating miRNAs and clinical parameters in a human cohort. (**B**) Circulating *miR-22-3p* levels are increased in mice after 15 weeks of a HFD but not a SAC diet. (**C**) Correlation between circulating *miR-22-3p* and fatty liver index (FLI) in humans. (**D**–**G**): Individuals with FLI > 60 and high *miR-22-3p* levels (High22) show equivalent body mass index (BMI) (**D**) but increased fasting plasma insulin (FPI) (**E**), fasting plasma glucose (FPG) (**F**), and homeostatic model assessment-insulin resistance (HOMA-IR) (**G**) than those with FLI > 60, but low *miR-22-3p* levels (Low22). n = 40 (**A**,**C**–**G**), n = 3/group (**B**). * *p* < 0.05 and *** *p* < 0.005 with respect to CT or FLI < 60 group. + *p* < 0.05 and ++ *p* < 0.01 with respect to High22 group. chol = cholesterol; G120 = glucose at 120 min during oral GTT; HDL = high density lipoprotein; LDL = low density lipoprotein; WHR = waist/hip ratio.

In a previous circulating miRNA analysis of a human cohort that included overweight/obese subjects with different degrees of glucose tolerance, we did not observe significant differences for any of the miRNAs that we have identified in HFD and SAC mice in the present study when the patients were analyzed according to glycemic state [23]. We decided to reanalyze the data focusing on those miRNAs upregulated in either HFD or SAC mice and perform correlation analyses with different clinical parameters (Figure 7A). *miR-122-5p* and *miR-223-3p* were increased in the plasma exosomes of both HFD and SAC mice, whereas *miR-378-3p*, *miR-101-3p*, *miR-107-3p*, and *miR-19-3p* were increased in HFD mice. SAC upregulated *miR-200b-3p* and *miR-322-5p* were not measured in the human profile. In addition, we included *miR-22-3p*, which is differentially regulated in the liver exosomes from both experimental groups. Interestingly, *miR-378-3p* showed significant positive correlations with a worse clinical outcome, whereas *miR-223-3p* evidenced significant negative correlations, suggestive of a protective role, in accordance with our previous data showing a decreased level of this miRNA in prediabetic subjects in a different cohort [28]. Moreover, *miR-122-5p* significantly correlated with TG levels and *miR-107-3p* with HDL. Surprisingly, *miR-22-3p* showed a strong correlation with most clinical values analyzed (Supplementary Table S12). As mentioned, *miR-22-3p* was the only miRNA showing inconsistent regulation between conditions, being downregulated in SAC liver exosomes and upregulated in HFD. Interestingly, although after 6 weeks of diet *miR-22-3p* was unchanged in plasma exosomes, our previous data showed that it is highly upregulated after 15 weeks of a HFD [13]. Circulating miRNA analysis of CT, HFD, and SAC mice confirmed that *miR-22-3p* is significantly increased in HFD plasma but unchanged in SAC mice after 15 weeks of diet (Figure 7B).

Hence, we analyzed the potential of circulating *miR-22-3p* as a biomarker for obesity-derived liver damage in humans. Remarkably, by distributing the subjects of the cohort according to their *miR-22-3p* levels, we observed a close association with the diagnosis of the patients ascribed to each group, with the lowest *miR-22-3p* levels including 90% of normoglycemic subjects (Supplementary Figure S7A). Moreover, by distributing the patients according to the FLI, a surrogate marker of hepatic steatosis [24], we observed a tendency for *miR-22-3p* to be increased in the steatosis-positive FLI > 60 group (Figure 7C, Supplementary Table S13). However, we noticed a high dispersion for *miR-22-3p* values in the FLI > 60 group, so we could separate this group into two subgroups: those with high (High22) and low (Low22) *miR-22-3p* (Supplementary Figure S7B). Both subgroups had an equivalent body mass index (BMI) and FLI (Figure 7D, Supplementary Figure S7C), but individuals with a high FLI and high *miR-22-3p* had increased fasting insulin and glucose, and HOMA-IR (Figure 7E–G), as well as a more unfavorable lipid profile (Supplementary Figure S7D). Indeed, ROC analysis indicates that the levels of circulating *miR-22-3p* in patients with FLI > 60 are a good biomarker (AUC = 0.849) to separate those subjects with HOMA-IR > 2.7 (Supplementary Figure S7E).

Overall, these data suggest that high circulating *miR-22-3p* may be used to identify those patients with a high FLI that may undergo a worse clinical evolution.

## 4. Discussion

The widespread incidence of obesity and the associated metabolic diseases has become a global health problem [29]. Here, hoping to obtain new insights in the etiopathogenesis of metabolic disease and identify novel biomarkers, we studied the effect of two different obesogenic diets on mouse metabolism, and the role of exo-miRNAs in the development of the pathogenic phenotypes. Interestingly, both a fat-rich diet and excess sucrose intake resulted in obesity in the long term, with both phenotypes almost indistinguishable in biometric and biochemical analyses. This situation mirrors the phenotypic similarity of diabetic patients, which masks different underlying pathological defects [2,3,4]. Body weight, fat distribution, and glycemia were comparable by 15 weeks, with the only difference being insulin levels, which although higher than the control in both experimental groups, were more elevated in HFD mice, probably due to the higher percentage of fat in the diet, as dietary fats amplify insulin secretion [30]. Interestingly, glucose tolerance was positively correlated with body weight, suggesting a more important role for obesity than hepatic steatosis in the global regulation of glucose metabolism [13].

Even though the excess intake of fat or sucrose both led to similar degrees of hepatic steatosis, liver transcriptomics evidenced that the pathways leading to it are different, with sucrose ingestion increasing lipid biosynthesis, whereas a HFD increased lipid storage. Calorimetry analysis further supported the increased DNL in SAC mice, which was accompanied by higher EE, thus providing a mechanism to explain the delayed weight gain in these mice. DNL is an energetically costly process that has been suggested to exert a protective effect upon weight gain [26,31]. Moreover, we observed that DNL was also required to maintain glucose homeostasis in SAC mice after glucose intake, as inhibition of FASN activity resulted in deregulated glycemia. FASN, whose expression was increased only in SAC mice, is a central regulator of both lipid and glucose metabolism [32]. The eWAT of SAC mice also showed increased expression of lipid biosynthesis genes, whereas HFD mice, which showed enlarged fat pads with adipocyte hypertrophy, displayed inflammatory pathway enrichment.

We and others have shown that exo-miRNAs carry information relevant to metabolic regulation, and manipulation of their abundance can lead to beneficial or pathological effects [13,14,16,17,18,33]. Here, by injecting siEGFP-transfected exosomes to an EGFP transgenic mouse, we further demonstrated that nucleic acids exogenously administered through exosomes can reach most tissues and exert a measurable phenotypic effect. The effect of CT, HFD, and SAC exosomes was comparable in all tissues studied, suggesting that exosomes can spread throughout the body. However, these data do not discard the existence of specific exosome subpopulations with different targeting capacities, as has been demonstrated for tumor-derived exosomes [34].

Treatment with HFD and SAC exosomes partially phenocopied some donor mice features, with iHFD mice showing increased adiposity, as well as being glucose intolerant and insulin resistant, as we had previously described when injecting plasma exosomes from long-term HFD mice [13]. The most surprising result was the increased expression of fructose handling enzymes in the liver of iSAC mice. These transcriptomic changes were functional, resulting in increased glucose and TG production after fructose ingestion. Interestingly, both iHFD and iSAC mice displayed hepatic steatosis associated with different gene expression profiles and metabolic outcomes. Indeed, injection of HFD and SAC plasma exosomes induced the most transcriptomic changes in eWAT and liver, respectively. This may be explained by the existence of the abovementioned exosome subpopulations, by the different exosomal cargo of both experimental models, or by the different response to that cargo by the recipient cells.

Specific plasma exo-miRNA changes in HFD and SAC mice may reflect a response to the respective environmental insults, whereas the common regulation may be a response to the common metabolic alterations observed, such as hepatic steatosis. Interestingly, from the six upregulated plasma exo-miRNAs in HFD mice after 6 weeks, three remained upregulated after 15 weeks [13], indicative of an early response, whereas the other three were only transiently upregulated.

We identified some miRNAs that were equally regulated in liver and plasma exosomes from HFD and SAC mice. However, the relationship is not as straightforward as expected. The quantity of exo-miRNAs that a tissue contributes to the general circulation may depend on the changes in the total mass of the tissue, the number of exosomes released by that tissue in each condition, or the content of a specific miRNA per exosome. In addition, the contribution of one tissue may be diluted by modifications in the input by other tissues. Moreover, some authors have postulated that the majority of exosomes in blood are released by hematopoietic cells, with tissue-derived exosomes representing a small percentage [35]. All these confounders preclude a direct correlation between the liver and plasma exo-miRNA data.

One striking result was the opposite regulation of *miR-22-3p* in liver exosomes from HFD and SAC mice. Increased *miR-22-3p* was also observed in the plasma of HFD mice after 15 weeks of diet [13], but the increase was absent in SAC mice. Interestingly, by studying a human cohort with different degrees of glucose tolerance according to American Diabetes Association established criteria [23,36], we observed that *miR-22-3p* correlated with most clinical parameters reflective of glucose and lipid metabolism. In our analysis, subjects with FLI < 60, indicative of a low probability of hepatic steatosis [24], have low *miR-22-3p*, but subjects with FLI > 60 displayed a high dispersion of *miR-22-3p* values, allowing their classification in two subgroups. Convergence of FLI > 60 and high *miR-22-3p* identifies the group with the worst clinical profile. The increased insulin, glucose, and HOMA-IR of the High22 group mirrors the obesity-driven phenotype of HFD mice, whereas the presence of hepatic steatosis without major alterations in glucose and lipid metabolism in the Low22 group is similar to the phenotype of SAC mice. Notably, the role of *miR-22-3p* in MAFLD has been intensely discussed, with reports showing both beneficial and pathological effects [37,38]. Our data is in accordance with *miR-22-3p* overexpression resulting in increased GNG in mice [39]. Moreover, *miR-22-3p* is considered a good metabolic target, as it regulates the genes involved in glucose and lipid metabolism, and inhibition in the control mice decreased circulating glucose and improved insulin sensitivity by activating the pathways related to lipid metabolism in both liver and eWAT and increasing EE [40]. Furthermore, *miR-22-3p* knockout mice also present increased EE [41], hence resulting in slower weight gain and improved metabolic parameters, although without decreasing hepatic steatosis [41,42]. Hence, circulating *miR-22-3p* could be used as a biomarker to identify two populations of patients with steatosis who may differ in their etiology, although further studies are required to confirm this.

In summary, we have shown that both increased dietary fat and high sucrose intake lead to hepatic steatosis through different metabolic pathways. Plasma exo-miRNA profiles differ between HFD and SAC mice, and injection into control mice reproduces some features of donor mice, suggesting that exosomes help the organism to adapt to environmental challenges.

## 5. Conclusions

We have shown, using metabolic and transcriptomic analyses, that both short-term high-fat or high-sucrose diets generate hepatic steatosis by activating different pathways. Furthermore, we have demonstrated, with calorimetric studies, that mice fed with a high-sucrose diet rely on de novo lipogenesis to maintain glucose homeostasis.

Moreover, we used a transgenic fluorescent mouse model to provide evidence that exogenous exosomes are not only widely distributed throughout the body tissues, but also induce phenotypically measurable effects. In this regard, the injection of plasma exosomes from HFD and SAC mice, with different plasma exo-miRNA profiles, reproduced some transcriptomic and metabolic features of the respective donor mice.

Finally, we identify circulating *miR-22-3p* as a candidate biomarker for MAFLD patient stratification.

## Data Availability

Microarray data have been deposited in the Gene Expression Omnibus (GEO) database (GSE202845 and GSE202854).

## Supplementary Materials

The following supporting information can be downloaded at: https://www.mdpi.com/article/10.3390/cells12010169/s1, Supplementary Methods, Table S1. Modified pathways identified by enrichment analysis of differentially expressed genes in liver of HFD or SAC mice as compared with CT mice; Table S2. Modified pathways identified by enrichment analysis of differentially expressed genes in eWAT of HFD or SAC mice as compared with CT mice; Table S3. Modified pathways identified by enrichment analysis of differentially expressed genes in gastrocnemius muscle of HFD or SAC mice as compared with CT mice; Table S4. Modified pathways identified by enrichment analysis of differentially expressed genes in liver of mice injected with plasma exosomes from HFD or SAC mice as compared with mice injected with plasma exosomes of CT mice; Table S5. Modified pathways identified by enrichment analysis of differentially expressed genes in eWAT of mice injected with plasma exosomes from HFD or SAC mice as compared with mice injected with plasma exosomes of CT mice; Table S6. Modified pathways identified by enrichment analysis of differentially expressed genes in gastrocnemius muscle of mice injected with plasma exosomes from HFD or SAC mice as compared with mice injected with plasma exosomes of CT mice; Table S7. miRNAs differentially expressed in exosomes isolated from plasma of HFD as compared with CT mice; Table S8. miRNAs differentially expressed in exosomes isolated from plasma of SAC as compared with CT mice; Table S9. miRNAs differentially expressed in exosomes isolated from liver as compared with liver tissue in CT mice; Table S10. miRNAs differentially expressed in exosomes isolated from liver of HFD as compared with CT mice; Table S11. miRNAs differentially expressed in exosomes isolated from liver of SAC as compared with CT mice; Table S12. Correlation coefficients between clinical parameters and selected circulating miRNAs in a human cohort; Table S13. Main clinical features and miR-22-3 p values of the study population; Figure S1. High-fat and high-sucrose diets lead to a convergent obese phenotype over time.; Figure S2. HFD and SAC induce distinct gene expression profiles in eWAT and liver.; Figure S3. SAC mice rely on DNL to maintain glucose homeostasis.; Figure S4. Injection of HFD and SAC plasma exosomes reproduce transcriptomic changes observed in donor mice.; Figure S5. Injection of HFD and SAC plasma exosomes reproduce the metabolic phenotype observed in donor mice.; Figure S6. The miRNA profile differs between HFD and SAC plasma and liver exosomes.; Figure S7. Circulating *miR-22-3p* can be used as a biomarker for MAFLD stratification.
