# Supplementary material for: Exosomes from Short-Term High-Fat or High-Sucrose Fed Mice Induce Hepatic Steatosis through Different Pathways
Source: Cells. 2022 Dec 31;12(1):169. doi: 10.3390/cells12010169 (PMC9818966; doi:10.3390/cells12010169)
Supplement: Supplementary file 1 [file cells-12-00169-s001.zip › cells-2116187-supplementary.pdf]

cells

Supplementary Information for

Exosomes from short-time high-fat or high-sucrose fed mice induce hepatic steatosis through different pathways

Carlos Castaño [1,2], Anna Novials [1,2,*] and Marcelina Párrizas [2,*]

[1] Instituto de Investigaciones Biomédicas August Pi i Sunyer (IDIBAPS), Barcelona-08036 (Spain)

[2] Centro de Investigación Biomédica en Red de Diabetes y Enfermedades Metabólicas (CIBERDEM), Barcelona-08036 (Spain)

* Correspondence: anovials@recerca.clinic.cat (A.N.); badcell@gmail.com (M.P.)

This PDF file includes

Supplementary Methods
Tables S1 to S13
Figures S1 to S7

Supplementary Methods

Animal studies

All *in vivo* studies were performed at the School of Medicine Animal Facilities (University of Barcelona). Procedures were conducted in accordance with principles of laboratory animal care (European and local government guidelines) and approved by the Animal Research Committee of the University of Barcelona (register number: 46/18). C57BL/6J male mice were used throughout the study. Mice were fed standard chow with either tap water or tap water supplemented with 50 % food-grade sucrose, or a high-fat diet (45 % calories from fat, *Open-Source Diets* #D12451) for the periods indicated below. Food and drink intake and animal weight were controlled weekly. Glucose tolerance test was performed by i.p. injection of 2 g/Kg glucose after 6 h fasting. Insulin sensibility was determined by i.p. injection of 0.5 U/Kg insulin after 6 h fasting. Pyruvate tolerance test was performed by i.p. injection of 1 g/Kg pyruvate after o/n fasting. Fructose tolerance test was performed by oral administration of 4g/Kg fructose after 6 h fasting. In all cases, tail blood glucose was measured at 0, 15, 30, 60 and 120 min using reactive strips in a *NovaPro* glucometer (1, 2). Circulating triglyceride levels were measured in tail blood with reactive strips using an *Accutrend* GCT glucometer after 6 h fasting and at 0 and 60 min during the fructose tolerance test. Plasma was obtained from 50 µl of tail blood centrifuged at 1,500 xg for 20 min (4ºC) in microvette EDTA tubes (*Sarstedt*). Free fatty acids were measured from 5 µl plasma after 6 h fasting with NEFA HR(2) Assay Kit (*Wako chemicals*). β-hydroxybutyrate levels were measured from 30 µl plasma after o/n fasting with Amplite Colorimetric Beta-Hydroxybutyrate Ketone Body Assay Kit (*AAT Bioquest*). Alanine Aminotransferase activity was measured from 10 µl plasma after 6h fasting with ALT/SGPT Activity Assay Kit (*Biovision*). Insulin levels were determined from 5 µl plasma at the 0- and 15-min time points during the glucose tolerance test and at the 0- and 30-min points during the pyruvate tolerance test with Mouse Insulin ELISA Kit (*Alpha Diagnostic International*). The HOMA-IR index was calculated as ($Insulin_{T0}$x$Glucose_{T0}$)/405, with glucose in mg/dL and insulin in mIU/L. Fat distribution was analyzed at the Experimental Magnetic Resonance Imaging Unit of the IDIBAPS. Briefly, mice were anesthetized by isoflurane inhalation and placed in supine position on a methacrylate bed equipped with a temperature maintenance system using a hot water circuit. Imaging technique is standardized and lasts around 30 min per mouse, using BioSpec 70/30 horizontal animal scanner (3). Adipose areas were quantified with ImageJ FIJI. At the sacrifice, liver, eWAT, sWAT and gastrocnemius muscle were dissected, weighed and flash-frozen in liquid N_2 for posterior protein and RNA analyses. Tibia length was measured with a caliber. Hepatic free fatty acid and triglyceride contents were measured after extraction with 3 M KOH and 65% ethanol with the NEFA-HR(2) Kit and the GPO-POD Colorimetric Kit (*Spinreact*) respectively as described (1, 2).

Experimental models

<u>DONORS:</u> 15-week-old male mice were fed standard chow (CT), standard chow with drinking water supplemented with 50% sucrose (SAC), or a high-fat diet (HFD) for 6 or 15 weeks. Four independent cohorts (n=6/group) were analyzed at the 6-weeks' time point and two more at the 15-weeks' time point (n=4/group). <u>INJECTED</u>: A first cohort of control male mice fed standard chow were injected i.v. through the tail vein with 100 µl PBS containing 200 µg exosomes isolated from plasma of CT (iCT), SAC (iSAC) or HFD (iHFD) mice and sacrificed 72h afterwards (acute treatment, n=4/group). A second cohort received a first i.v. injection of 100 µg exosomes and two more injections of 50 µg each, separated by 4 days (chronic treatment, n=4/group). Metabolic analyses were performed starting 15 days after the first injection. <u>EGFP:</u> For biodistribution studies, transgenic male mice overexpressing EGFP (*Jackson*) (4) were injected i.v. with 100 µl PBS containing 50 µg exosomes isolated from plasma of CT, HFD or SAC mice and transfected with 500 pmol siEGFP (*Thermo Fisher Scientific*). As a positive control, i.v. injection of 500 pmol siEGFP coupled to Invivofectamine 3.0 was performed in parallel (n=3/group). EGFP fluorescence was analyzed *in vivo* with an IVIS Imaging System and mice were sacrificed 72 h after injection for microscopy analysis of the tissues of interest in a TCS SPE confocal microscope. In addition, 100 mg of the indicated tissues were lysed in 500 µl RIPA lysis buffer and 100 µl were measured in a Tecan Fluorimeter with 480 nm excitation, 515 nm emission and a gain of 70.

Indirect calorimetry

Gas exchange during the glucose and fructose tolerance tests was measured in a closed one-lane treadmill with the CaloSys apparatus TSE Systems. Each mouse was individually placed in the stationary treadmill chamber without inclination for 5 min to allow for gas equilibration. Chamber was then opened, mice were administered with the specified glucose or fructose doses through oral gavage and immediately replaced in the chamber. VO_2 and VCO_2 measurements were continued for the next 50 min. From these values, the respiratory exchange ratio was determined as VCO_2/VO_2, carbohydrate oxidation was determined as $4.55xVCO_2-3.21VO_2$, and energy expenditure was determined as $(VO_2x3.94+VCO_2x1.11)/1.44$ (5). For a set of experiments, mice were pretreated with an i.p injection of 10 mg/Kg FASN inhibitor C75 (*Sigma*) 1h before starting the test (6). At the end of each experiment, glucose and triglycerides were measured with reactive strips.

Exosome isolation, characterization, and transfection

Exosomes were isolated from 500 µl mouse plasma obtained by cardiac puncture at sacrifice. Briefly, plasma was diluted with an equal volume of PBS and samples were sequentially centrifuged at 2,000 xg for 30 min (4ºC), 10,000 xg for 45 min (4ºC), filtered through a 0.22 µm syringe filter and ultracentrifuged o/n at 120,000 xg (4ºC) using a S110AT Rotor in a Sorvall MX 150 ultracentrifuge. Pellets were resuspended in PBS and ultracentrifuged again at 120,000 xg for 3 h (4ºC). The final pellets were resuspended in 100 µl PBS.

Interstitial liver exosomes were isolated as previously described by us and others (2, 7). Briefly, CT, HFD and SAC mice were perfused through the heart at the left ventricle with 10 ml PBS supplemented with 0.5 mM EGTA at a rate of 2.5 ml/min for 4 min to remove blood from the tissues. EGTA was washed out with a second perfusion of 10 ml PBS supplemented with 0.5% BSA. Mice were finally perfused under the same settings with 10 ml 0.5 mg/ml collagenase IV (*Sigma-Aldrich*) in HBSS (*Sigma-Aldrich*) maintained at 37ºC. Liver was dissected and finely minced with forceps to avoid cell rupture. Digestion was stopped by adding 8 ml PBS with BSA and tissue suspension was centrifuged at 100 xg for 5 min twice to precipitate undigested material. Cleared supernatants were further processed to isolate exosomes by following the protocol described for plasma samples. After the 2,000 xg and 10,000 xg centrifugations, supernatants were further cleaned by size exclusion chromatography using SmartSEC columns (*SBI Biosciences*). Samples were then filtered through a 0.22 µm syringe filter and ultracentrifuged as described. The final pellets containing the liver interstitial exosomes were resuspended in 100 µl PBS.

Total exosome protein was quantified by Bradford Assay (*Sigma-Aldrich*) and complementary analyses by measuring the acetylcholinesterase activity known to be associated with exosomes were also performed in 5 µl of exosomes in suspension with Exocet Exosome Quantification Assay Kit (*System Biosciences*). Vesicle morphology was analyzed by negative staining in a Transmission Electron Microscope. Briefly, 30 µl exosomes diluted 1/10 to 1/20 with PBS were allowed to dry on top of formvar carbon-coated grids for 25 min and contrasted with 2 % uranyl acetate for 2 min. Preparations were observed in a JEOL 1010 100 kV TEM. Particle number and diameter was determined by Nanoparticle Tracking Analysis with NanoSight LM10 equipment using different dilutions (1/10 to 1/50) and the following parameters: camera at 30 frames per second, camera level at 16, temperature between 21–25°C and video recording time 60 s. Nanosight Software analyzed raw data videos by triplicate. For western blotting of exosome membrane markers, 10 µl exosomes were resolved by 12% SDS-PAGE and transferred to a NTC membrane (*Whatman*). Anti-CD63 (H-193) (sc-15363, *Santa Cruz Biotechnology*) and anti-CD9 (EPR2949) (ab92726, *Abcam*) were diluted 1/250 and 1/500 respectively in TBS (20mM Tris, 150mM NaCl, pH7.5) supplemented with 5% BSA and visualized by blotting with HRP-conjugated secondary anti-rabbit antibody (NA934V, *GE Healthcare Bio-Sciences*). Chemiluminescence was detected by using the ECL Plus Reagents (*GE Healthcare Bio-Sciences*), in a LAS4000 Lumi-Imager.

Exosomes (50 µg) were transfected with 500 pmol siEGFP by using Exo-Fect Exosome Transfection Reagent (*System Biosciences*) as described (1, 2).

Histochemistry and immunofluorescence

Tissues were fixed with 4 % PFA o/n. Liver was equilibrated in 30 % sucrose and embedded in OCT, whereas eWAT was embedded in paraffin. 10 μm sections were obtained with a CM 1950 cryostat or a RM2135 microtome respectively and applied to poly-lysine coated slides. Oil Red O and Hematoxylin & Eosin stainings were performed by following the protocols at IHCWorld (http://www.ihcworld.com). Liver sections were mounted in Aquatex mounting media (*Merck*) and adipose sections were mounted in Pertex (*HistoLab)*. Images were obtained with a BX41TF trinocular microscope. Adipocyte size and number were determined from the Hematoxylin & Eosin- stained sections using ImageJ FIJI. To study biodistribution of siEGFP-transfected exosomes and siEGFP-Invivofectamine complexes, liver and gastrocnemius muscle were embedded in OCT to obtain 10 μm sections, whereas eWAT was finely minced with scissors and applied to a slide. Nuclei were stained with 1:500 Hoestch in PBS for 3 min and samples were mounted with Dako fluorescence mounting medium. Images were collected using TCS SPE confocal microscope.

Western blotting

Total protein was extracted using RIPA lysis buffer from frozen tissue samples after powdering with a ceramic mortar and pestle in liquid N_2. Protease and phosphatase inhibitors were added fresh to the lysis buffer. 50 μg quantified by Bradford Assay (*Sigma-Aldich*) were resolved by 7.5 % Mini-PROTEAN TGX Precast Protein Gels (*Bio-Rad*) and transferred to a NTC membrane (*Whatman*). After 5% BSA blocking, rabbit anti-ACACA (ab72046), anti-FASN (ab22759) (both *Abcam*) and anti-TUBBA (T6074) (*Sigma*) antibodies were diluted 1:1000 in 1% TBS-BSA and visualized by blotting with 1:5000 HRP-conjugated secondary anti-rabbit or anti-mouse antibody (both *GE Healthcare*). Chemiluminiscence was detected with Pierce ECL Western Blotting Substrate (*Thermo Fisher Scientific*) in a LAS4000 Lumi-Imager.

RNA isolation and gene and miRNA expression analysis

Total RNA was extracted from frozen liver, eWAT and gastrocnemius muscle with miRNeasy Mini Kit (*Qiagen*), treated with DNAse, and quantified by absorbance with Nanodrop. 250 ng RNA was used to analyze size and integrity in RNA TapeStation 4200. 150 ng good quality RNA samples (RIN >9) with a ratio 260/280=1.8-2 were used for microarray hybridization. Fragmentation and Biotin labelling of ss-cDNA was prepared according to Affymetrix WT PLUS Reagent Kit user guide, using an automated system (Biomek FX System). Next, ss-cDNA was hybridized for 17hr at 45ºC on Clariom™ S HT, mouse array plate, using the automated GeneTitan System, which includes the hybridization oven, Fluidic Station and Scanner. We analyzed 3 tissues in 6 groups of mice (CT, HFD, SAC, iCT, iHFD and iSAC) for a total of n=18 types of samples, performing biological quadruplicates for a total of n=72 microarray hybridizations. Microarray hybridization data was analyzed with Transcriptome Analysis Console 4.0 (*Applied Biosystems*) using RMA analysis. PCAs, volcano plots, hierarchical clustering and pathway enrichment analysis were performed with the same software. Heatmaps of pathways identified by enrichment analysis of genes were created with heatmapper.ca. Venn's diagrams for comparing up lists of genes were created with Venny 2.1 (https://bioinfogp.cnb.csic.es).

For exosomal miRNA analysis, total RNA was extracted from 25 μl isolated plasma exosomes or liver interstitial exosomes with miRNeasy Mini Kit (*Qiagen*). Exosomes used for RNA isolation were pretreated with 0.1 μg/μl RNAse A for 40 min at 37ºC to eliminate contamination from cellular rupture, particularly in the case of interstitial exosomes. RNAse digestion was stopped by addition of RNAsin (*Promega Corporation*). An equal volume of each RNA sample was retrotranscribed with mircury LNA™ Universal RT microRNA PCR (*Qiagen*). Profiling of 375 miRNAs was performed by Real time RT-PCR using predesigned panels with LNA specific primers (*Qiagen*) in a 7900HT Fast Thermocycler (*Applied Biosystems*). Differential expression was determined with GenEx Software v.6 (*Exiqon*) after normalization to the global mean Ct of the plate.

Clinical cohort

All participants gave written informed consent, and the studies were approved by the Research and Ethics Committees of the Hospital Clinic, University of Barcelona (register 2011/6945). A total of 40 serum samples from well-characterized male individuals with different degrees of glucose tolerance according to American Diabetes Association established criteria (8) were selected from among those stored in the Hospital Clínic-IDIBAPS Biobank: 13 control subjects, 10 prediabetic individuals with isolated impaired fasting glucose, 9 prediabetic subjects with isolated impaired glucose tolerance, and 8 newly diagnosed T2D patients. Circulating miRNAs were extracted from 200 µl serum with the miRNeasy kit and analyzed by real-time RT-PCR using 384-well panels with primers specific for the 176 miRNAs most commonly found in plasma/serum (*Qiagen*), in a Roche LC480 II thermocycler. Differential expression was analyzed with GenEx software (*Exiqon*) by normalizing to the mean Ct of the whole plate. Results obtained by analyzing the samples according to glycemic state were published in (9). For the current study, subjects were distributed according to the fatty liver index, calculated as described (9, 10), and circulating *miR-22-3p* values: control group (FLI<60), hepatic steatosis with high *miR-22-3p* (High22) and hepatic steatosis with low *miR-22-3p* (Low22). Additional information regarding inclusion criteria and biochemical parameter measurement can be found in (9). Heatmap of correlation coefficients was created with heatmapper.ca. Receiver Operating Characteristic curves were plotted using ROC Station and the area under the curve was calculated to determine discrimination accuracy.

Statistics

Differences between groups were determined by either t-test analysis when only two groups were compared or by One-way ANOVA with t-test analysis for the pair wise comparison of 3 or more groups with different number of values. The Shapiro Wilk test p-value was performed to ascertain normality of the samples. Symbols indicate significance with respect to each control group, unless otherwise indicated.

References

1. Castaño C, et al. Obesity-associated exosomal miRNAs modulate glucose and lipid metabolism in mice. *Proc. Natl. Acad. Sci. U.S.A.* **2018**, *115*, 12158–12163.
2. Castaño C, et al. Delivery of muscle-derived exosomal miRNAs induced by HIIT improves insulin sensitivity through down-regulation of hepatic FoxO1 in mice. *Proc. Natl. Acad. Sci. U.S.A.* **2020**, *117*, 30335-30343.
3. García-Eguren G, et al. Chronic hypercortisolism causes more persistent visceral adiposity than HFD-induced obesity. *J. Endocrinol.* **2019**, *242*, 65-77.
4. Okabe M, et al. Green mice as a source of ubiquitous green cells. *FEBS Lett.* **1997**, *19407*, 313-9.
5. Frayn KN. Calculation of substrate oxidation rates in vivo from gaseous exchange. *J. Appl. Physiol. Respir. Environ. Exerc. Physiol.* **1983**, *55*, 628-34.
6. Cavalcanti-de-albuquerque JP et al. Regulation of substrate utilization and adiposity by Agrp neurons. *Nat. Commun.* **2019**, *10*, 311.
7. Crewe C, et al. An Endothelial-to-Adipocyte Extracellular Vesicle Axis Governed by Metabolic State. *Cell.* **2018**, *175*, 695-708.
8. American Diabetes Association. Diagnosis and Classification of Diabetes Mellitus. *Diabetes Care.* **2020**, *43*, S14-S31.
9. Párrizas M, et al. Circulating miR-192 and miR-193b are markers of prediabetes and are modulated by an exercise intervention. *J. Clin. Endocrinol. Metab.* **2015**, *100*, E407–E415.
10. Bedogni G, et al. The Fatty Liver Index: a simple and accurate predictor of hepatic steatosis in the general population. *BMC Gastroenterol*. **2006**, 6, 33.

Table S1. Modified pathways identified by enrichment analysis of differentially expressed genes in liver of HFD or SAC mice as compared with CT mice.

LIVER HFD vs CT					
**Pathway**	**Up**	**Representative Up genes**	**Down**	**Representative Down genes**	**p-value**
Cholesterol biosynthesis	0	-	13	*Sqle,Idi1*	0.0000
Cholesterol metabolism	1	*Fads2*	17	*Hmgcr,Mvk*	0.0000
PPAR signaling pathway	4	*Fabp4,Cpt1a*	7	*Acsl3,Fabp5*	0.0000
Oxidation by CYP450	3	*Cyp21a1*	4	*Cyp51,Cyp7a1*	0.0001
Statin pathway	2	*Apoc2,Apoa4*	2	*Cyp7a1,Hmgcr*	0.0024
Retinol metabolism	3	*Abcg8,Abcg5*	2	*Retsat,Crabp2*	0.0052
ω3/ω6 fatty acid synthesis	1	*Fads2*	2	*Pla2g5,Acsl3*	0.0123
Eicosanoid metabolism	0	-	3	*Cyp2c37*	0.0123
Fatty acid beta-oxidation	3	*Fabp2,Fabp4*	1	*Acsl3*	0.0134
Type II interferon signaling	2	*Tap1,Cxcl9*	2	*Socs3,Icam1*	0.0165
Fatty acid biosynthesis	0	-	3	*Acsl3,Acacb*	0.0251
Endochondral ossification	3	*Igf2,Frzb*	2	*Hmgcs1,Sox9*	0.0342
p53 signaling	3	*Cdkn1a,Thbs1*	2	*Gadd45g,Sesn1*	0.0480
**LIVER SAC vs CT**					
**Pathway**	**Up**	**Representative Up genes**	**Down**	**Representative Down genes**	**p-value**
ω9 fatty acid synthesis	6	*Fasn,Elovl5*	0	-	0.0000
Fatty acid biosynthesis	7	*Acsl5,Acaca*	0	-	0.0000
Glycolysis	7	*Eno3,Slc2a5*	1	*Slc2a1*	0.0000
Pentose phosphate pathway	4	*G6pdx,Rpia*	0	-	0.0000
Cholesterol metabolism	9	*Hmgcr,Fads1*	0	-	0.0002
Eicosanoid metabolism	1	*Cyp2c38*	4	*Cyp2c44*	0.0002
Complement activation	0	-	5	*C8a,C2*	0.0002
Glycolysis & gluconeogenesis	7	*Gpi1,Pklr*	1	*Slc2a1*	0.0003
Striated muscle contraction	6	*Myl1,Acta1*	1	*Neb*	0.0008
mRNA processing	1	*Oas1a*	4	*Eif4e,Elavl3*	0.0014
Cholesterol biosynthesis	4	*Fdps,Mvd*	0	-	0.00143
Elongation of VLCFA	4	*Elovl5,Fads1*	0	-	0.0018
PPAR signaling pathway	9	*Acaa1b,Acsl5*	0	-	0.0021
ω3/ω6 fatty acid synthesis	4	*Fads2,Acot2*	0	-	0.0023
Oxidation by CYPP450	3	*Cyp8b1*	3	*Cyp26a1*	0.0024
Statin pathway	4	*Apoc2,Apoa4*	0	-	0.0044
Steroid biosynthesis	1	*Cyp17a1*	2	*Hsd3b4,F13b*	0.0091
Ala and asp metabolism	1	*Pcx*	2	*Ass1,Agxt*	0.0137
Oxidative stress	5	*G6pdx,Slc6a9*	3	*Gsta1,Idh2*	0.0144
TCA cycle	3	*Pdk4,Pdk1*	1	*Idh2*	0.0213
Leptin-insulin signaling	1	*Lepr*	2	*Socs3,Socs2*	0.0228
Glutathione metabolism	3	*Gstt1,G6pdx*	0	-	0.0264
Urea cycle	0	-	3	*Ass1,Oat*	0.0303
Fatty acid beta-oxidation	4	*Acsl5,Acss2*	0	-	0.0318
Triacylglyceride synthesis	2	*Gpam,Mogat1*	1	*Agpat5*	0.0436

Table S2. Modified pathways identified by enrichment analysis of differentially expressed genes in eWAT of HFD or SAC mice as compared with CT mice.

**eWAT HFD vs CT**					
**Pathway**	**Up**	**Representative Up genes**	**Down**	**Representative Down genes**	**p-value**
B cell receptor signaling	57	*Grb2,Arpc2*	9	*Foxo1,Cr2*	0.0000
Complement and coagulation	7	*C1qa,C3ar1*	28	*Fgb,C9*	0.0000
Focal adhesion	45	*Col4a1,Rac2*	24	*Met,Egfr*	0.0000
Oxidative stress pathway	8	*Cd44,Hpgds*	30	*Ptgs1,Sod1*	0.0000
Cell cycle	30	*E2f2,Plk1*	6	*Hdac2,Cdh1*	0.0000
Chemokine signaling	50	*Cx3cr1,Arrb2*	13	*Pik3r1,Gnb5*	0.0000
TCA cycle	0	-	17	*Pdha1,Pdhb*	0.0000
T cell receptor signaling	44	*Map4k1,Was*	3	*Tuba4a,Ppp3cb*	0.0000
Electron transport chain	2	*Atpif1,Ucp2*	35	*Cox6c,Atp5*	0.0000
Ribosomal proteins	2	*Rps6ka1*	1	*Rpl36*	0.0001
Mapk signaling pathway	28	*Stmn1,Ptpn7*	27	*Akt2,Ngf*	0.0001
Adipogenesis genes	18	*Ddit3,Cebpb*	27	*Retn,Adipoq*	0.0001
PPAR signaling pathway	9	*Fabp7,Cpt1b*	22	*Pparg,Apoa1*	0.0001
p53 signaling	21	*Chek1,Bax*	6	*Mdm4,Perp*	0.0002
Eicosanoid synthesis	3	*Tbxas1,Ltc4s*	8	*Ptges2,Dpep1*	0.0003
Glucuronidation	0	-	10	*Ugp2,Pgm3*	0.0004
Apoptosis	21	*Casp9,Bak1*	8	*Birc2,Irf6*	0.0006
Glycolysis	3	*Tpi1,Hk3*	14	*Slc2a4,Slc2a5*	0.0006
Macrophage markers	5	*Cd83,Cd68*	2	*F3,Cd163*	0.0008
Estrogen metabolism	0	-	8	*Ugt1a2,Gstm1*	0.00114
Fatty acid biosynthesis	0	-	11	*Fasn,Acaca*	0.0014
Insulin signaling	23	*Pik3cd,Arf6*	25	*Irs1,Irs2*	0.0019
Statin pathway	4	*Pltp,Ldlr*	6	*Apoc3,Abca1*	0.0023
Glycogen metabolism	3	*Gys2,Ppp2r5b*	11	*Pygm,Gys1*	0.0040
Fatty acid beta-oxidation	4	*Tpi1,Cpt1a*	9	*Acadm,Pnpla2*	0.0171
Toll-like receptor signaling	24	*Tlr1,Tlr9*	5	*Pik3r1,Irf3*	0.0212
IL-6 signaling pathway	20	*Il6,Fes*	9	*Gab1,Ncoa1*	0.0222
Prostaglandin synthesis	7	*Anxa5,Anxa1*	4	*Ptgis,Hpgd*	0.0394
**eWAT SAC vs CT**					
**Pathway**	**Up**	**Representative Up genes**	**Down**	**Representative Down genes**	**p-value**
Ribosomal proteins	0	-	40	*Rps18,Rpl3*	0.0000
Cholesterol biosynthesis	10	*Fdps,Sqle*	1	*Fdft1*	0.0000
Cholesterol metabolism	14	*Dhcr7,Sc5d*	3	*Hmgcs2,Fdft1*	0.0000
Fatty acid beta-oxidation	5	*Acss2,Cpt1a*	4	*Acadl,Crat*	0.0014
Fatty acid biosynthesis	3	*Acss2,Acly*	3	*Echdc3,Ech1*	0.0063
Oxidative stress	3	*G6pdx,Pgd*	12	*Ggt1,Sod1*	0.0065
LCFA β-oxidation	2	*Cpt1a,Acsl3*	3	*Acadl,Acadm*	0.0067
Complement activation	1	*Masp1*	4	*C2,C3,C9*	0.0089
Glycolysis	5	*Pklr,Aldoa*	3	*Slc2a3,Pdp1*	0.0091
ω9 fatty acid synthesis	4	*Acsl3,Elovl6*	0	-	0.0212
p53 signaling	6	*Cdkn1a,Casp9*	5	*Rrm2b,Sesn1*	0.0217
Retinol metabolism	2	*Rara,Scarb1*	5	*Aldh1a3,Rbp1*	0.0329
Oxidation by CYP450	4	*Cyb5b,Cyb5r1*	3	*Cyp1b1,Cyp2e1*	0.0372
PPAR signaling pathway	6	*Scd2,Acsl3*	6	*Acsbg1,Acadl*	0.0379

Table S3. Modified pathways identified by enrichment analysis of differentially expressed genes in gastrocnemius muscle of HFD or SAC mice as compared with CT mice.

**Gas HFD vs CT**					
**Pathway**	**Up**	**Representative Up genes**	**Down**	**Representative Down genes**	**p-value**
Electron transport chain	35	*Cox6c,Atp5f1*	0	-	0.0000
Fatty acid beta-oxidation	16	*Cpt2,Acads*	2	*Lipf,Acsl3*	0.0000
Cholesterol metabolism	3	*Acat1,Acsl1*	18	*Srebf1,Abca1*	0.0000
PPAR signaling pathway	10	*Cd36,Ppara*	14	*Adipoq,Plin1*	0.0000
Fatty acid biosynthesis	6	*Acsl1,Acaa2*	6	*Fasn,Acaca*	0.0000
Oxidative phosphorylation	19	*Ndufb9,Ndufs1*	0	-	0.0000
LCFA beta-oxidation	9	*Acadl,Acadm*	1	*Acsl3*	0.0000
ω9 fatty acid synthesis	2	*Acsl1,Acot2*	7	*Fads1,Fads2*	0.0000
Complement and coagulation	1	*Masp1*	16	*C1qa,Kng1*	0.0000
Statin pathway	1	*Lpl*	7	*Ldlr,Abca1*	0.0001
Glucuronidation	0	-	7	*Ugt1a5,Ugt2b1*	0.0001
Synthesis of ketone bodies	3	*Acat1,Oxct1*	1	*Hmgcs2*	0.0001
Estrogen metabolism	0	-	6	*Ugt1a10,Cyp1a*	0.0002
TCA cycle	8	*Pdk4,Sdhb*	1	*Pcx*	0.0004
ω3/ω6 fatty acid synthesis	2	*Acsl1,Acot2*	4	*Elovl5,Acsl3*	0.0013
Glycolysis & gluconeogenesis	4	*Dld,Ldhb*	7	*Pck1,Aldob*	0.0013
NRs in lipid metabolism	1	*Ppara*	7	*Abca1,Vdr*	0.0016
SREBF and cholesterol	1	*Ppara*	4	*Srebf1,Srebf2*	0.0021
**Gas SAC vs CT**					
**Pathway**	**Up**	**Representative Up genes**	**Down**	**Representative Down genes**	**p-value**
Electron transport chain	16	*Cox6b1,Uqcr10*	0	-	0.0000
Apoptosis	3	*Bad,Casp3*	8	*Irf1,Tnf*	0.0010
Oxidative phosphorylation	9	*Atp5g1,Ndufb2*	0	-	0.0011
Complement and coagulation	0	-	9	*Tfpi,C3*	0.0020
Cholesterol metabolism	0	-	7	*Hmgcr,Fads2*	0.0180
B cell receptor signaling	0	-	14	*Cd22,Vav2*	0.0183
Type II interferon signaling	0	-	5	*Irf4,Irf8*	0.0186
Glycerolipids	1	*Crls1*	3	*Dgat1,Pnpla2*	0.0195
Omega-9 fatty acid synthesis	0	-	3	*Elovl5,Fads2*	0.0241
TNF-alpha NF-kB signaling	2	*Casp3,Pebp1*	13	*Nfkbia,Nfkb2*	0.0312
Elongation of VLCFAs	0	-	3	*Elovl5,Fads1*	0.0346
Fatty acid ω-oxidation	0	-	2	*Cyp1a2,Cyp2e1*	0.0379
IL-5 signaling pathway	0	-	7	*Prkcb,Itgb2*	0.0386
Ω3/ω6 fatty acid synthesis	0	-	3	*Fads2,Fads1*	0.0406
Complement activation	0	-	3	*C3,C6,C4b*	0.0406
Toll-like receptor signaling	3	*Irf7,Tlr8*	6	*Cd40,Nfkb2*	0.0461
NRs in lipid metabolism	0	-	4	*Vdr,Nr1h4*	0.0470

Table S4. Modified pathways identified by enrichment analysis of differentially expressed genes in liver of mice injected with plasma exosomes from HFD or SAC mice as compared with mice injected with plasma exosomes of CT mice.

**LIVER iHFD vs iCT**					
**Pathway**	**Up**	**Representative Up genes**	**Down**	**Representative Down genes**	**p-value**
GPCRs, other	0	-	1	*Olfr48*	0.0018
mRNA processing	6	*Bicc1,Dhx8*	5	*Mettl3,Snrpd3*	0.0232
FAS pathway	2	*Mapkapk2*	3	*Casp8,Fas*	0.0247
Inflammatory response	2	*Fn1,Tnfrsf1b*	2	*Lamc2,Il5ra*	0.0413
**LIVER iSAC vs iCT**					
**Pathway**	**Up**	**Representative Up genes**	**Down**	**Representative Down genes**	**p-value**
Glycolysis & gluconeogenesis	5	*Slc2a5,G6pc*	1	*Eno3*	0.0322
Glycolysis	4	*Hk1,Pdk2*	1	*Eno3*	0.0346
IL-5 signaling pathway	5	*Foxo3,Itgb2*	2	*Il5ra,Sdcbp*	0.0479
GPCRs, other	4	*Cysltr1,Olfr24*	0	-	0.0496

Table S5. Modified pathways identified by enrichment analysis of differentially expressed genes in eWAT of mice injected with plasma exosomes from HFD or SAC mice as compared with mice injected with plasma exosomes of CT mice.

**eWAT iHFD vs iCT**					
**Pathway**	**Up**	**Representative Up genes**	**Down**	**Representative Down genes**	**p-value**
mRNA processing	5	*Prpf4,Col4a3*	7	*Rngtt,Snrpb2*	0.0002
Eicosanoid synthesis	5	*Ptgds,Ggt1*	1	*Pnpla8*	0.0008
Oxylipins	6	*Alox8,Alox12*	0	-	0.0019
Prostaglandin synthesis	1	*Ptgds*	5	*Anxa5,Anxa1*	0.0120
Elongation of VLCFAs	4	*Fads2,Elovl4*	0	-	0.0158
ω3/ω6 fatty acid synthesis	4	*Fads1,Acox3*	0	-	0.01965
Pluripotency	6	*Tcf7,Nr6a*	3	*P4ha1,Nr0b1*	0.0206
Focal adhesion	11	*Mylk,Araf*	9	*Col4a1,Fyn*	0.0309
T cell receptor signaling	1	*Plcg1*	2	*Cd247,Fyn*	0.0490
**eWAT iSAC vs iCT**					
**Pathway**	**Up**	**Representative Up genes**	**Down**	**Representative Down genes**	**p-value**
Ala and asp metabolism	2	*Asl,Agxt*	0	-	0.0436

Table S6. Modified pathways identified by enrichment analysis of differentially expressed genes in gastrocnemius muscle of mice injected with plasma exosomes from HFD or SAC mice as compared with mice injected with plasma exosomes of CT mice.

Gas iHFD vs iCT					
**Pathway**	**Up**	**Representative Up genes**	**Down**	**Representative Down genes**	**p-value**
mRNA processing	3	*Zbp1,Col4a3*	2	*Cpsf1,Snrpa1*	0.0089
Nucleotide metabolism	2	*Mthfd2,Polb*	1	*Polg*	0.0179
Blood clotting cascade	0	-	3	*Serpinf2,Plg*	0.0206
Statin pathway	0	-	3	*Apoc3,Apoa1*	0.0206
G1 to S cell cycle control	3	*Cdkn2d,Atm*	2	*Cdk6,Rpa3*	0.0373
**Gas iSAC vs iCT**					
**Pathway**	**Up**	**Representative Up genes**	**Down**	**Representative Down genes**	**p-value**
Striated muscle contraction	6	Mybpc1,Myh7	0	-	0.0017
mRNA processing	3	Adar,Col4a3	1	Dazl	0.0033
B cell receptor signaling	7	Dok1,Nfatc2	4	Ptpn18,Ccne1	0.0055
Inflammatory response	4	Col1a1,Col1a2	0	-	0.0102
Fatty acid omega-oxidation	0	-	2	Cyp1a1,Cyp2e1	0.0157
Focal adhesion	10	Thbs4,Itga9	1	Pgf	0.0427

Table S7. miRNAs differentially expressed in exosomes isolated from plasma of HFD as compared with CT mice (n=3/group).

(PCT) vs (PHFD)	Fold change	Difference (A-B log scale)	P-Value
miR-122-5p	-8.11	-3.02	**0.0321**
miR-101b-3p	-4.19	-2.07	**0.0438**
miR-378a-3p	-1.88	-0.91	**0.0033**
miR-19b-3p	-1.77	-0.82	**0.0482**
miR-107-3p	-1.74	-0.80	**0.0122**
miR-223-3p	-1.56	-0.64	**0.0092**
miR-23b-3p	1.47	0.55	**0.0495**
RNU1A1	1.91	0.93	**0.0228**
RNU5G	2.11	1.08	**0.0198**
miR-130b-3p	2.84	1.50	**0.0403**
miR-199a-5p	2.95	1.56	**0.0050**
miR-200c-3p	1.66	0.73	0.0548
miR-7a-5p	2.66	1.41	0.0584
miR-24-3p	1.47	0.56	0.0647
miR-100-5p	1.86	0.90	0.0695
miR-151-5p	2.60	1.38	0.0728
miR-296-5p	-1.83	-0.87	0.0881
miR-375-3p	2.17	1.12	0.1076
rno-miR-223-3p	-1.60	-0.68	0.1102
miR-339-5p	1.51	0.59	0.1169
miR-18a-5p	-2.84	-1.51	0.1173
rno-miR-143-3p	-2.01	-1.01	0.1237
miR-219a-5p	-1.71	-0.78	0.1419
miR-484	-2.16	-1.11	0.1420
miR-297a-5p	1.73	0.79	0.1438
miR-365-3p	-3.71	-1.89	0.1513
miR-10b-5p	1.95	0.96	0.1562
miR-30e-5p	1.86	0.89	0.1663
rno-miR-214-3p	1.98	0.99	0.1683
miR-106b-5p	-1.54	-0.62	0.1746
miR-652-3p	-2.09	-1.07	0.1811
miR-15a-5p	1.47	0.56	0.1928
miR-210-3p	-1.68	-0.75	0.2044
miR-142-5p	1.72	0.78	0.2046
miR-148a-3p	-2.47	-1.31	0.2085
miR-324-5p	2.00	1.00	0.2127
let-7a-5p	1.57	0.65	0.2132
miR-350-3p	-2.37	-1.25	0.2226
miR-142-3p	1.71	0.77	0.2247
miR-328-3p	1.38	0.46	0.2296
miR-451a	-1.75	-0.81	0.2332
miR-500-3p	2.63	1.40	0.2419
miR-140-5p	1.50	0.58	0.2440
miR-16-5p	-1.23	-0.29	0.2444
miR-425-5p	1.45	0.54	0.2562
miR-467a-5p	1.42	0.50	0.2662
miR-19a-3p	-3.11	-1.64	0.2711

miR-532-5p	-2.01	-1.01	0.2713
miR-151-3p	2.47	1.30	0.2727
let-7g-5p	-1.27	-0.35	0.2775
miR-21a-5p	-1.20	-0.26	0.2783
let-7d-5p	2.02	1.02	0.2840
miR-34a-5p	1.69	0.76	0.2930
miR-30c-5p	1.47	0.55	0.2951
let-7c-5p	1.24	0.31	0.2954
miR-146a-5p	1.40	0.48	0.3004
let-7f-5p	1.46	0.54	0.3070
miR-423-5p	1.64	0.71	0.3178
miR-101a-3p	-1.50	-0.59	0.3203
miR-150-5p	1.21	0.28	0.3285
let-7e-5p	2.66	1.41	0.3305
miR-212-3p	1.82	0.87	0.3615
miR-29b-3p	-1.09	-0.13	0.3697
miR-322-5p	-1.30	-0.38	0.3756
miR-497-5p	-1.19	-0.25	0.3864
miR-145a-5p	1.53	0.62	0.4015
miR-574-3p	1.60	0.68	0.4056
miR-3107-5p	-1.24	-0.31	0.4103
U6 snRNA	2.69	1.43	0.4148
rno-miR-338-3p	-1.58	-0.66	0.4273
miR-361-5p	-1.39	-0.47	0.4311
let-7b-5p	-1.30	-0.38	0.4331
miR-195a-5p	1.52	0.61	0.4493
miR-25-3p	-1.38	-0.47	0.4546
miR-30d-5p	-1.49	-0.58	0.4680
miR-30b-5p	1.25	0.33	0.4804
miR-191-5p	-1.26	-0.34	0.4842
miR-193b-3p	1.56	0.64	0.4880
miR-152-3p	-1.66	-0.73	0.5147
miR-125b-5p	1.21	0.28	0.5305
miR-22-3p	-1.18	-0.24	0.5314
miR-103-3p	1.44	0.53	0.5351
miR-326-3p	-1.15	-0.20	0.5365
miR-29c-3p	-1.19	-0.25	0.5579
miR-20a-5p	-1.28	-0.35	0.5625
miR-194-5p	-1.41	-0.49	0.5645
miR-130a-3p	-1.21	-0.27	0.5716
miR-99a-5p	1.36	0.44	0.5727
miR-320-3p	1.33	0.41	0.5772
miR-192-5p	-1.31	-0.39	0.5967
miR-222-3p	-1.64	-0.71	0.5992
miR-23a-3p	-1.11	-0.15	0.6212
miR-125a-5p	-1.21	-0.27	0.6507
miR-188-5p	-1.36	-0.45	0.6563
miR-143-3p	-1.22	-0.29	0.6641
miR-214-3p	1.13	0.18	0.7077
miR-301a-3p	1.19	0.25	0.7254
miR-27b-3p	-1.20	-0.27	0.7297

miR-205-5p	-1.11	-0.15	0.7385
miR-423-3p	-1.05	-0.07	0.7524
miR-93-5p	1.12	0.17	0.7534
miR-1a-3p	-1.14	-0.19	0.7576
miR-203-3p	-1.16	-0.22	0.7924
miR-200b-3p	-1.19	-0.25	0.7927
miR-139-5p	1.07	0.10	0.8047
miR-141-3p	1.10	0.14	0.8063
miR-148b-3p	-1.16	-0.21	0.8101
miR-362-3p	-1.15	-0.21	0.8365
miR-199a-3p	1.06	0.08	0.8532
miR-27a-3p	1.05	0.08	0.8595
miR-17-5p	1.05	0.08	0.8665
let-7i-5p	1.04	0.06	0.8854
miR-33-5p	1.06	0.08	0.8855
miR-132-3p	-1.09	-0.13	0.9113
rno-miR-345-5p	1.09	0.13	0.9155
miR-126a-3p	1.02	0.03	0.9225
miR-133b-3p	-1.03	-0.04	0.9488
miR-29a-3p	1.00	0.01	0.9491
miR-133a-3p	-1.03	-0.04	0.9550
miR-15b-5p	1.02	0.03	0.9623
miR-144-3p	-1.01	-0.01	0.9873
miR-342-3p	-1.00	0.00	1.0000
miR-185-5p	1.00	0.00	1.0000

Table S8. miRNAs differentially expressed in exosomes isolated from plasma of SAC as compared with CT mice (n=3/group).

(PCT) vs (PHFD)	Fold change	Difference (A-B log scale)	P-Value
miR-200b-3p	-3.69	-1.88	**0.0306**
miR-122-5p	-2.86	-1.52	**0.0298**
miR-322-5p	-2.39	-1.25	**0.0177**
miR-223-3p	-1.42	-0.50	**0.0208**
miR-29b-3p	1.42	0.51	**0.0242**
miR-23b-3p	1.50	0.58	**0.0464**
miR-301a-3p	1.66	0.73	**0.0414**
miR-100-5p	2.09	1.06	**0.0191**
miR-339-5p	2.19	1.13	**0.0118**
miR-142-5p	2.21	1.14	**0.0145**
miR-151-3p	2.87	1.52	**0.0381**
miR-199a-5p	3.39	1.76	**0.0333**
miR-467a-5p	4.04	2.01	**0.0076**
miR-101a-3p	-2.86	-1.51	0.0542
miR-130a-3p	1.55	0.63	0.0604
let-7c-5p	1.55	0.63	0.0652
rno-miR-223-3p	-1.60	-0.68	0.0653
miR-194-5p	-2.91	-1.54	0.0675
miR-151-5p	1.99	0.99	0.0710
miR-423-5p	2.23	1.15	0.0740
miR-7a-5p	1.84	0.88	0.0759
miR-425-5p	1.68	0.75	0.0782
miR-30b-5p	2.50	1.32	0.0808
miR-141-3p	-1.68	-0.75	0.0887
miR-143-3p	-2.14	-1.10	0.0942
miR-3107-5p	-1.77	-0.83	0.1082
miR-203-3p	-2.92	-1.55	0.1141
let-7a-5p	1.81	0.85	0.1207
miR-152-3p	-1.82	-0.86	0.1317
miR-326-3p	1.59	0.67	0.1383
miR-17-5p	-1.48	-0.57	0.1397
miR-212-3p	2.62	1.39	0.1521
miR-365-3p	-2.04	-1.03	0.1531
miR-423-3p	-1.40	-0.49	0.1716
miR-324-5p	1.92	0.94	0.1738
miR-30c-5p	1.75	0.81	0.1830
rno-miR-143-3p	-1.90	-0.93	0.1853
miR-142-3p	1.82	0.86	0.1870
miR-19b-3p	-1.24	-0.31	0.1887
miR-16-5p	-1.63	-0.70	0.1898
miR-205-5p	-1.78	-0.83	0.1957
miR-139-5p	1.35	0.43	0.1979
miR-574-3p	-2.00	-1.00	0.2016
miR-106b-5p	-1.50	-0.59	0.2035
miR-133a-3p	1.75	0.81	0.2203
miR-107-3p	-2.10	-1.07	0.2242
miR-532-5p	2.05	1.03	0.2274

miR-101b-3p	-1.67	-0.74	0.2287
let-7g-5p	1.36	0.44	0.2302
miR-210-3p	-1.76	-0.81	0.2427
let-7d-5p	1.80	0.85	0.2539
miR-296-5p	1.45	0.53	0.2618
miR-19a-3p	-3.37	-1.75	0.2678
miR-103-3p	2.31	1.21	0.2961
miR-24-3p	1.22	0.29	0.2968
miR-146a-5p	1.33	0.41	0.3008
miR-200c-3p	2.24	1.16	0.3051
miR-27b-3p	-1.54	-0.62	0.3103
let-7e-5p	2.86	1.52	0.3179
miR-25-3p	-1.69	-0.75	0.3251
miR-29c-3p	-1.22	-0.29	0.3352
U6 snRNA	3.44	1.78	0.3394
miR-350-3p	-1.48	-0.57	0.3482
miR-93-5p	-1.28	-0.35	0.3592
miR-29a-3p	1.10	0.14	0.3668
miR-27a-3p	1.73	0.79	0.3777
miR-378a-3p	1.06	0.09	0.3928
miR-193b-3p	1.77	0.82	0.3956
miR-185-5p	1.68	0.75	0.3977
miR-22-3p	-1.29	-0.36	0.4071
miR-484	-1.39	-0.48	0.4173
miR-500-3p	1.79	0.84	0.4247
miR-23a-3p	-1.18	-0.24	0.4324
miR-20a-5p	-1.36	-0.45	0.4490
miR-30e-5p	1.36	0.45	0.4587
RNU5G	1.33	0.41	0.4641
miR-195a-5p	1.43	0.52	0.4789
miR-34a-5p	-1.21	-0.27	0.4906
miR-126a-3p	-1.12	-0.17	0.4908
miR-10b-5p	1.49	0.57	0.4945
miR-132-3p	1.37	0.46	0.4960
miR-214-3p	-1.17	-0.22	0.5013
miR-297a-5p	1.21	0.27	0.5038
miR-150-5p	1.15	0.20	0.5262
miR-320-3p	1.48	0.56	0.5620
rno-miR-214-3p	1.28	0.35	0.5625
miR-30d-5p	-1.33	-0.41	0.5748
miR-148a-3p	1.28	0.35	0.5822
let-7f-5p	1.31	0.39	0.5900
miR-451a	-1.31	-0.39	0.5949
miR-497-5p	-1.22	-0.29	0.5992
miR-99a-5p	-1.27	-0.34	0.6243
rno-miR-338-3p	1.19	0.25	0.6254
miR-18a-5p	-1.42	-0.50	0.6293
miR-133b-3p	1.19	0.25	0.6881
miR-199a-3p	-1.23	-0.30	0.6928
miR-361-5p	1.13	0.18	0.7168
miR-1a-3p	1.15	0.20	0.7208

miR-192-5p	-1.08	-0.11	0.7397
miR-130b-3p	1.20	0.26	0.7462
miR-362-3p	-1.31	-0.39	0.7472
miR-328-3p	1.06	0.09	0.7487
miR-140-5p	-1.17	-0.23	0.7728
miR-652-3p	-1.29	-0.36	0.7788
miR-144-3p	-1.10	-0.14	0.7895
miR-188-5p	1.16	0.22	0.7967
miR-145a-5p	1.08	0.11	0.8013
rno-miR-345-5p	-1.20	-0.26	0.8144
miR-33-5p	-1.08	-0.11	0.8264
miR-342-3p	-1.07	-0.10	0.8451
miR-148b-3p	1.07	0.10	0.8503
miR-222-3p	1.17	0.23	0.8644
let-7b-5p	1.03	0.05	0.8957
miR-21a-5p	-1.02	-0.03	0.9012
miR-15a-5p	1.03	0.05	0.9080
miR-219a-5p	1.04	0.05	0.9110
miR-125b-5p	1.03	0.04	0.9216
miR-125a-5p	1.02	0.03	0.9639
let-7i-5p	1.01	0.01	0.9745
miR-191-5p	1.01	0.01	0.9863
RNU1A1	1.01	0.01	0.9877
miR-15b-5p	-1.01	-0.01	0.9881
miR-375-3p	1.00	0.01	0.9895

Table S9. miRNAs differentially expressed in exosomes isolated from liver as compared with liver tissue in CT mice (exosomes n=4, tissue n=3).

(Exosomes) vs (Tissue)	Fold change	Difference (A-B log scale)	P-Value
RNU1A1	-25.94	-4.70	**0.0182**
miR-144-3p	-15.55	-3.96	**0.0010**
miR-339-5p	-12.50	-3.64	**0.0007**
RNU5G	-12.48	-3.64	**0.0104**
miR-125a-5p	-9.45	-3.24	**0.0008**
miR-212-3p	-9.45	-3.24	**0.0188**
U6 snRNA	-9.29	-3.22	**0.0007**
miR-200c-3p	-9.29	-3.22	**0.0019**
miR-331-3p	-8.82	-3.14	**0.0058**
miR-107-3p	-7.61	-2.93	**0.0022**
miR-3107-5p	-7.04	-2.82	**0.0013**
rno-miR-146b-5p	-6.96	-2.80	**0.0272**
miR-484	-6.36	-2.67	**0.0080**
miR-451a	-6.33	-2.66	**0.0008**
miR-141-3p	-5.59	-2.48	**0.0142**
miR-139-5p	-5.57	-2.48	**0.0038**
miR-149-5p	-5.12	-2.36	**0.0277**
miR-379-5p	-4.79	-2.26	**0.0140**
miR-26a-5p	-4.76	-2.25	**0.0244**
rno-miR-223-3p	-4.40	-2.14	**0.0023**
miR-142-3p	-4.39	-2.14	**0.0022**
miR-31-5p	-4.36	-2.12	**0.0013**
let-7e-5p	-4.31	-2.11	**0.0022**
miR-30b-5p	-4.20	-2.07	**0.0018**
rno-miR-200b-3p	-3.91	-1.97	**0.0364**
miR-28a-5p	-3.80	-1.93	**0.0004**
let-7a-5p	-3.80	-1.92	**0.0028**
miR-551b-3p	-3.73	-1.90	**0.0429**
let-7f-5p	-3.71	-1.89	**0.0038**
miR-374b-5p	-3.64	-1.86	**0.0173**
miR-30c-5p	-3.55	-1.83	**0.0022**
miR-103-3p	-3.39	-1.76	**0.0026**
miR-150-5p	-3.25	-1.70	**0.0052**
rno-miR-450a-5p	-3.05	-1.61	**0.0101**
miR-99b-5p	-3.04	-1.60	**0.0011**
let-7c-5p	-2.90	-1.54	**0.0050**
miR-223-3p	-2.90	-1.53	**1E-04**
let-7d-5p	-2.83	-1.50	**0.0049**
miR-214-3p	-2.64	-1.40	**0.0286**
miR-145a-5p	-2.53	-1.34	**0.0180**
miR-98-5p	-2.49	-1.31	**0.0096**
let-7g-5p	-2.47	-1.31	**0.0030**
miR-203-3p	-2.32	-1.21	**0.0033**
miR-429-3p	-2.25	-1.17	**0.0219**
miR-142-5p	-2.25	-1.17	**0.0221**
miR-181a-5p	-2.19	-1.13	**0.0081**
miR-342-3p	-2.19	-1.13	**0.0051**

miR-423-3p	-2.19	-1.13	**0.0138**
miR-125b-5p	-2.00	-1.00	**0.0039**
miR-23b-3p	-1.82	-0.87	**0.0017**
miR-192-5p	-1.56	-0.65	**0.0382**
miR-425-5p	-1.22	-0.29	**0.0453**
miR-15b-5p	1.54	0.62	**0.0057**
miR-100-5p	1.55	0.63	**0.0040**
miR-143-3p	1.65	0.72	**0.0084**
miR-27a-3p	1.81	0.85	**0.0293**
miR-378a-3p	2.12	1.08	**0.0011**
miR-199a-3p	2.17	1.12	**0.0014**
miR-30a-5p	2.21	1.14	**0.0288**
miR-29a-3p	2.35	1.24	**0.0258**
miR-365-3p	2.38	1.25	**0.0499**
miR-185-5p	2.43	1.28	**0.0257**
rno-miR-338-3p	2.43	1.28	**0.0479**
miR-382-5p	2.87	1.52	**0.0407**
miR-15a-5p	2.94	1.56	**0.0008**
miR-322-5p	3.01	1.59	**0.0025**
miR-423-5p	3.01	1.59	**0.0021**
miR-146a-5p	3.08	1.62	**0.0118**
miR-23a-3p	3.28	1.71	**0.0002**
miR-101a-3p	3.50	1.81	**0.0004**
miR-676-3p	3.72	1.90	**0.0020**
miR-27b-3p	3.79	1.92	**0.0002**
miR-132-3p	3.81	1.93	**0.0009**
miR-532-5p	4.03	2.01	**0.0039**
let-7a-1-3p	4.03	2.01	**0.0081**
miR-122-5p	4.11	2.04	**0.0017**
miR-362-3p	4.43	2.15	**0.0428**
miR-25-3p	4.52	2.18	**0.0002**
miR-10b-5p	4.74	2.24	**0.0014**
miR-324-3p	4.79	2.26	**7E-05**
miR-21a-5p	4.81	2.27	**3E-05**
miR-101b-3p	5.14	2.36	**3E-06**
miR-320-3p	5.45	2.45	**0.0020**
miR-201-5p	5.61	2.49	**0.0156**
miR-34a-5p	5.74	2.52	**0.0004**
miR-222-3p	6.18	2.63	**0.0001**
miR-497-5p	6.22	2.64	**0.0013**
miR-335-5p	6.28	2.65	**4E-05**
miR-361-5p	7.95	2.99	**0.0036**
miR-130a-3p	8.64	3.11	**7E-05**
miR-22-3p	9.48	3.24	**5E-06**
miR-210-3p	9.73	3.28	**0.0044**
miR-152-3p	10.18	3.35	**1E-06**
miR-221-3p	11.37	3.51	**8E-05**
miR-148b-3p	15.37	3.94	**4E-05**
miR-148a-3p	18.53	4.21	**8E-08**
miR-592-5p	22.22	4.47	**0.0063**
miR-7a-5p	2.73	1.45	0.0508

let-7i-5p	1.96	0.97	0.0508
miR-218-5p	-2.50	-1.32	0.0509
miR-18a-5p	-2.91	-1.54	0.0528
miR-106b-5p	-2.16	-1.11	0.0536
miR-18a-3p	3.18	1.67	0.0537
miR-187-3p	-2.20	-1.14	0.0552
miR-26b-5p	-2.62	-1.39	0.0606
miR-99a-5p	1.45	0.53	0.0634
miR-29c-3p	3.03	1.60	0.0706
rno-miR-143-3p	1.39	0.48	0.0710
miR-455-5p	-2.19	-1.13	0.0720
rno-miR-200c-3p	-3.92	-1.97	0.0729
miR-350-3p	-3.89	-1.96	0.0765
miR-351-5p	-5.98	-2.58	0.0786
miR-194-5p	1.44	0.52	0.0810
miR-190a-5p	-2.77	-1.47	0.0894
miR-376c-3p	-3.50	-1.81	0.0975
miR-151-3p	1.88	0.91	0.0983
miR-200a-3p	-1.94	-0.95	0.1090
miR-375-3p	-2.55	-1.35	0.1138
miR-328-3p	4.59	2.20	0.1160
miR-191-5p	-1.58	-0.66	0.1174
miR-193a-3p	2.53	1.34	0.1217
miR-501-3p	2.22	1.15	0.1219
miR-30e-5p	1.80	0.85	0.1219
miR-296-5p	-3.60	-1.85	0.1239
miR-183-5p	-2.42	-1.27	0.1427
miR-154-5p	1.37	0.46	0.1455
miR-19a-3p	1.88	0.91	0.1484
miR-219a-5p	2.54	1.35	0.1542
miR-20a-3p	-3.64	-1.86	0.1556
miR-30d-5p	-1.22	-0.29	0.1557
miR-345-5p	-1.97	-0.97	0.1684
miR-93-5p	-1.35	-0.43	0.1696
miR-874-3p	1.52	0.60	0.1723
miR-128-3p	-3.64	-1.86	0.1758
miR-130b-3p	1.96	0.97	0.1843
miR-188-5p	2.22	1.15	0.1955
miR-434-3p	-2.06	-1.04	0.1986
miR-181b-5p	-1.58	-0.66	0.2052
miR-450a-5p	-1.36	-0.44	0.2214
let-7b-5p	-1.61	-0.69	0.2232
miR-137-3p	1.61	0.68	0.2386
miR-1a-3p	3.00	1.59	0.2395
miR-133b-3p	2.43	1.28	0.2595
rno-miR-551b-3p	-1.67	-0.74	0.2654
miR-151-5p	-1.36	-0.45	0.2749
miR-32-5p	2.23	1.16	0.2765
miR-195a-5p	-1.22	-0.28	0.2767
miR-16-5p	-1.56	-0.64	0.2817
miR-200b-3p	-1.24	-0.31	0.2932

rno-miR-214-3p	-1.79	-0.84	0.2984
miR-17-5p	-1.88	-0.91	0.2985
miR-199a-5p	-1.41	-0.50	0.3277
miR-338-3p	1.67	0.74	0.3282
rno-miR-136-5p	2.50	1.32	0.3319
miR-30c-2-3p	-2.19	-1.13	0.3362
miR-421-3p	2.05	1.04	0.3371
miR-182-5p	-1.75	-0.81	0.3438
miR-301a-3p	-1.73	-0.79	0.3547
miR-339-3p	-1.69	-0.76	0.3907
miR-106a-5p	-1.57	-0.65	0.3914
miR-140-5p	1.19	0.25	0.4202
miR-33-5p	-1.71	-0.78	0.4227
rno-miR-340-3p	-1.67	-0.74	0.4578
miR-652-3p	-1.25	-0.32	0.4674
miR-126a-3p	-1.19	-0.26	0.5131
miR-324-5p	1.50	0.59	0.5169
rno-miR-345-5p	1.26	0.34	0.5537
miR-96-5p	1.20	0.27	0.5918
miR-133a-3p	1.60	0.68	0.6104
miR-503-5p	1.28	0.35	0.6187
miR-500-3p	-1.15	-0.20	0.6836
miR-19b-3p	1.17	0.23	0.7439
miR-136-5p	1.21	0.28	0.7631
miR-411-5p	-1.26	-0.33	0.7859
miR-24-3p	1.06	0.08	0.7967
miR-193b-3p	1.12	0.17	0.8004
miR-574-3p	-1.07	-0.09	0.8069
miR-467a-5p	-1.20	-0.26	0.8104
miR-217-5p	-1.08	-0.12	0.8254
miR-326-3p	1.12	0.17	0.8634
miR-802-5p	-1.04	-0.06	0.8906
miR-139-3p	1.09	0.13	0.9098
miR-20a-5p	1.03	0.05	0.9148
miR-297a-5p	-1.05	-0.07	0.9228
miR-340-5p	1.04	0.06	0.9460
miR-29b-3p	1.06	0.08	0.9479

Table S10. miRNAs differentially expressed in exosomes isolated from liver of HFD as compared with CT mice (n=4/group).

(LCT) vs (LHFD)	Fold change	Difference (A-B log scale)	P-Value
miR-34a-5p	-2.73	-1.45	**0.0000**
miR-497-5p	-1.88	-0.91	**0.0381**
miR-676-3p	-1.67	-0.74	**0.0380**
miR-152-3p	-1.52	-0.60	**0.0317**
miR-15b-5p	-1.49	-0.57	**0.0104**
miR-148a-3p	-1.47	-0.56	**0.0239**
miR-22-3p	-1.37	-0.46	**0.0457**
miR-378a-3p	-1.36	-0.44	**0.0008**
miR-23b-3p	1.31	0.38	**0.0195**
miR-93-5p	1.60	0.68	**0.0456**
miR-143-3p	1.66	0.73	**0.0059**
miR-30d-5p	1.70	0.76	**0.0098**
miR-99b-5p	1.74	0.80	**0.0486**
miR-30a-5p	1.78	0.84	**0.0484**
miR-181a-5p	1.94	0.95	**0.0107**
miR-200b-3p	1.95	0.96	**0.0250**
miR-150-5p	3.16	1.66	**0.0454**
miR-450a-5p	3.35	1.74	**0.0137**
rno-miR-143-3p	1.50	0.58	0.0533
let-7g-5p	1.49	0.57	0.0563
rno-miR-223-3p	1.62	0.70	0.0637
miR-3107-5p	2.15	1.11	0.0638
miR-425-5p	1.36	0.44	0.0707
miR-15a-5p	-1.43	-0.51	0.0715
miR-142-5p	1.75	0.81	0.0725
miR-101b-3p	-1.23	-0.30	0.0734
miR-210-3p	-1.41	-0.49	0.0817
U6 snRNA	-1.98	-0.99	0.0821
miR-30c-5p	1.57	0.65	0.0827
miR-126a-3p	1.61	0.69	0.0863
miR-125b-5p	1.32	0.40	0.0882
rno-miR-338-3p	1.81	0.86	0.0917
miR-142-3p	1.61	0.69	0.0923
miR-199a-5p	1.58	0.66	0.1033
miR-30e-5p	1.71	0.78	0.1088
miR-193a-3p	3.04	1.60	0.1124
miR-146a-5p	1.61	0.69	0.1157
miR-503-5p	2.38	1.25	0.1212
miR-361-5p	-1.38	-0.46	0.1214
miR-122-5p	1.47	0.56	0.1241
miR-322-5p	-1.36	-0.44	0.1393
miR-148b-3p	-1.66	-0.73	0.1418
miR-99a-5p	-1.31	-0.39	0.1435
miR-194-5p	-1.24	-0.31	0.1657
miR-190a-5p	-1.62	-0.70	0.1770
miR-423-5p	-1.26	-0.34	0.1786
miR-342-3p	1.53	0.61	0.1809

miR-151-5p	1.36	0.45	0.1860
miR-185-5p	-1.38	-0.47	0.1950
miR-223-3p	1.28	0.35	0.2017
miR-21a-5p	1.24	0.32	0.2065
miR-532-5p	-1.34	-0.42	0.2066
let-7c-5p	1.33	0.41	0.2101
let-7f-5p	1.31	0.39	0.2153
miR-130b-3p	-1.70	-0.77	0.2216
miR-140-5p	1.23	0.30	0.2269
miR-17-5p	1.85	0.89	0.2310
miR-28a-5p	1.42	0.50	0.2409
miR-106b-5p	1.54	0.62	0.2410
miR-141-3p	-2.22	-1.15	0.2474
miR-423-3p	-12.76	-3.67	0.2580
miR-362-3p	1.82	0.86	0.2625
miR-429-3p	1.27	0.34	0.2707
miR-20a-5p	1.34	0.42	0.2756
miR-29b-3p	2.34	1.23	0.2791
let-7e-5p	1.35	0.44	0.2857
miR-144-3p	1.89	0.92	0.2867
miR-139-5p	1.49	0.57	0.3012
miR-31-5p	1.42	0.51	0.3082
miR-345-5p	-1.58	-0.66	0.3115
let-7a-5p	1.29	0.37	0.3149
miR-501-3p	1.45	0.54	0.3175
miR-222-3p	-1.12	-0.16	0.3200
miR-219a-5p	1.85	0.89	0.3247
miR-382-5p	1.60	0.68	0.3318
miR-335-5p	-1.42	-0.50	0.3343
miR-324-3p	1.21	0.27	0.3538
miR-10b-5p	-1.27	-0.35	0.3688
miR-191-5p	1.26	0.34	0.3716
miR-467a-5p	2.03	1.02	0.3820
miR-133a-3p	-2.18	-1.12	0.3850
let-7d-5p	1.26	0.33	0.3895
miR-16-5p	1.35	0.43	0.4120
miR-26b-5p	1.47	0.56	0.4137
miR-30b-5p	1.24	0.31	0.4218
miR-103-3p	1.22	0.28	0.4349
miR-137-3p	1.35	0.43	0.4414
miR-201-5p	1.41	0.49	0.4451
miR-100-5p	1.12	0.16	0.4560
miR-375-3p	-1.49	-0.58	0.4589
let-7a-1-3p	1.29	0.37	0.4641
miR-101a-3p	-1.10	-0.14	0.4824
miR-133b-3p	-1.56	-0.64	0.4934
miR-874-3p	-1.25	-0.32	0.4967
miR-199a-3p	1.12	0.16	0.4977
miR-324-5p	-1.41	-0.50	0.5024
miR-365-3p	-1.23	-0.30	0.5063
miR-203-3p	1.14	0.18	0.5156

miR-188-5p	-1.42	-0.51	0.5174
miR-26a-5p	1.28	0.36	0.5393
miR-98-5p	1.31	0.39	0.5411
miR-154-5p	1.41	0.49	0.5550
RNU1A1	-1.59	-0.67	0.5889
miR-339-5p	-1.32	-0.40	0.5913
miR-297a-5p	-1.30	-0.38	0.5975
miR-500-3p	1.24	0.31	0.6027
rno-miR-214-3p	-1.30	-0.37	0.6076
miR-214-3p	-1.14	-0.19	0.6296
miR-30c-2-3p	-1.35	-0.43	0.6366
miR-19b-3p	1.28	0.35	0.6401
miR-27a-3p	1.08	0.11	0.6627
miR-350-3p	1.26	0.34	0.6761
miR-29a-3p	-1.11	-0.15	0.6786
miR-32-5p	-1.32	-0.40	0.6787
miR-132-3p	-1.05	-0.07	0.6829
miR-200a-3p	1.19	0.25	0.6876
miR-421-3p	1.33	0.41	0.6978
miR-33-5p	1.36	0.44	0.7090
miR-326-3p	1.26	0.34	0.7154
miR-7a-5p	1.14	0.19	0.7164
miR-434-3p	1.22	0.29	0.7178
miR-25-3p	-1.09	-0.12	0.7198
miR-340-5p	-1.21	-0.27	0.7258
miR-455-5p	-1.11	-0.15	0.7278
miR-107-3p	-1.12	-0.17	0.7289
miR-221-3p	1.06	0.08	0.7570
miR-18a-3p	-1.14	-0.19	0.7643
rno-miR-345-5p	-1.14	-0.19	0.7645
miR-484	1.12	0.17	0.7662
miR-18a-5p	-1.15	-0.21	0.7695
let-7b-5p	1.10	0.13	0.7772
miR-592-5p	-1.06	-0.08	0.7816
miR-151-3p	1.05	0.08	0.7868
miR-192-5p	1.05	0.06	0.7965
miR-181b-5p	1.14	0.19	0.8011
RNU5G	1.17	0.23	0.8053
miR-802-5p	-1.06	-0.08	0.8109
miR-130a-3p	-1.05	-0.06	0.8218
miR-331-3p	-1.15	-0.20	0.8222
miR-23a-3p	1.04	0.06	0.8241
miR-24-3p	1.05	0.07	0.8474
miR-27b-3p	-1.02	-0.03	0.8632
miR-320-3p	-1.05	-0.06	0.8673
miR-338-3p	-1.09	-0.12	0.8731
miR-301a-3p	1.08	0.11	0.8846
miR-1a-3p	-1.06	-0.08	0.9300
miR-652-3p	-1.03	-0.04	0.9313
miR-125a-5p	-1.04	-0.05	0.9389
let-7i-5p	1.02	0.02	0.9456

miR-328-3p	1.05	0.07	0.9472
miR-193b-3p	-1.02	-0.03	0.9662
miR-195a-5p	-1.01	-0.01	0.9742
miR-187-3p	1.01	0.02	0.9743
miR-145a-5p	1.01	0.01	0.9750
miR-29c-3p	-1.01	-0.02	0.9800
miR-19a-3p	-1.01	-0.01	0.9882
miR-574-3p	1.00	0.01	0.9893
miR-200c-3p	-1.00	0.00	1.0000

Table S11. miRNAs differentially expressed in exosomes isolated from liver of SAC as compared with CT mice (n=4/group).

(LCT) vs (LSAC)	Fold change	Difference (A-B log scale)	P-Value
miR-190a-5p	-2.93	-1.55	**0.0332**
miR-214-3p	-2.39	-1.26	**0.0379**
miR-335-5p	-2.20	-1.14	**0.0134**
miR-34a-5p	-2.18	-1.13	**0.0031**
miR-223-3p	-1.62	-0.69	**0.0192**
miR-143-3p	1.55	0.63	**0.0097**
miR-27b-3p	1.78	0.83	**0.0209**
miR-324-3p	1.86	0.90	**0.0232**
miR-122-5p	1.93	0.94	**0.0355**
miR-221-3p	1.94	0.95	**0.0437**
miR-592-5p	2.04	1.03	**0.0435**
miR-22-3p	2.09	1.07	**0.0026**
miR-193a-3p	4.66	2.22	**0.0137**
rno-miR-223-3p	-2.18	-1.12	0.0514
miR-101b-3p	1.34	0.42	0.0577
miR-29a-3p	1.92	0.94	0.0601
miR-425-5p	1.57	0.65	0.0648
miR-30e-5p	2.19	1.13	0.0665
miR-31-5p	-1.72	-0.78	0.0679
miR-210-3p	1.54	0.62	0.0686
miR-200a-3p	-2.09	-1.06	0.0697
miR-365-3p	1.96	0.97	0.0698
rno-miR-338-3p	2.24	1.16	0.0736
miR-30b-5p	-1.67	-0.74	0.0747
miR-501-3p	2.31	1.21	0.0752
miR-133a-3p	-2.88	-1.53	0.0779
let-7f-5p	-1.48	-0.57	0.0789
miR-200c-3p	-2.49	-1.31	0.0801
rno-miR-143-3p	1.35	0.43	0.0811
miR-154-5p	-1.74	-0.80	0.0821
miR-142-3p	-1.66	-0.73	0.0847
miR-148b-3p	1.65	0.72	0.0905
miR-141-3p	-2.48	-1.31	0.0928
miR-362-3p	2.71	1.44	0.0969
let-7e-5p	-1.66	-0.73	0.1032
miR-107-3p	-1.86	-0.90	0.1088
miR-27a-3p	1.43	0.52	0.1192
miR-455-5p	-1.62	-0.69	0.1271
miR-99a-5p	1.37	0.45	0.1292
let-7d-5p	-1.45	-0.54	0.1339
miR-10b-5p	2.25	1.17	0.1369
miR-500-3p	1.69	0.75	0.1375
miR-125b-5p	1.35	0.43	0.1447
miR-18a-3p	2.18	1.12	0.1498
miR-193b-3p	1.73	0.79	0.1537
miR-15a-5p	1.30	0.38	0.1607
miR-28a-5p	-1.56	-0.65	0.1623

let-7g-5p	-1.30	-0.38	0.1624
miR-3107-5p	1.94	0.96	0.1648
miR-152-3p	-1.17	-0.23	0.1662
miR-181b-5p	-1.65	-0.72	0.1669
miR-802-5p	1.58	0.66	0.1802
miR-106b-5p	-1.53	-0.61	0.1814
miR-350-3p	-2.23	-1.16	0.1975
miR-15b-5p	1.44	0.53	0.2021
miR-24-3p	1.30	0.38	0.2071
miR-93-5p	1.34	0.42	0.2113
miR-133b-3p	-1.79	-0.84	0.2130
miR-130b-3p	-1.67	-0.74	0.2134
rno-miR-345-5p	1.62	0.69	0.2172
miR-125a-5p	-1.76	-0.82	0.2310
miR-297a-5p	-2.50	-1.32	0.2360
miR-29c-3p	1.86	0.90	0.2403
miR-26a-5p	-1.63	-0.70	0.2418
miR-301a-3p	-1.60	-0.68	0.2544
miR-188-5p	-2.01	-1.01	0.2548
miR-450a-5p	1.25	0.32	0.2641
miR-99b-5p	-1.54	-0.63	0.2713
miR-148a-3p	1.42	0.51	0.2796
miR-7a-5p	1.49	0.57	0.2803
miR-16-5p	1.50	0.59	0.2915
miR-423-5p	1.22	0.29	0.2955
miR-219a-5p	1.82	0.86	0.3051
miR-132-3p	1.33	0.41	0.3152
miR-434-3p	1.43	0.51	0.3266
miR-676-3p	1.47	0.55	0.3280
miR-222-3p	1.40	0.48	0.3333
miR-574-3p	-1.30	-0.37	0.3344
miR-199a-5p	-1.42	-0.50	0.3364
miR-324-5p	-1.71	-0.78	0.3404
miR-339-5p	-1.34	-0.42	0.3489
let-7a-5p	-1.29	-0.37	0.3514
miR-30c-5p	-1.26	-0.33	0.3857
U6 snRNA	-1.55	-0.63	0.3863
miR-142-5p	1.23	0.30	0.3865
miR-467a-5p	-1.84	-0.88	0.3919
miR-21a-5p	1.21	0.27	0.4030
miR-429-3p	-1.52	-0.61	0.4173
miR-33-5p	1.63	0.71	0.4282
miR-194-5p	1.14	0.19	0.4325
miR-151-5p	-1.19	-0.25	0.4470
miR-30a-5p	1.38	0.47	0.4504
miR-19a-3p	1.33	0.41	0.4862
miR-185-5p	1.20	0.27	0.5064
miR-98-5p	-1.25	-0.32	0.5112
miR-137-3p	1.31	0.38	0.5155
miR-103-3p	-1.17	-0.23	0.5310
miR-497-5p	-1.20	-0.26	0.5325

miR-421-3p	1.50	0.58	0.5389
miR-144-3p	1.37	0.45	0.5525
let-7c-5p	-1.14	-0.18	0.5529
miR-191-5p	-1.15	-0.20	0.5625
miR-342-3p	-1.14	-0.18	0.5647
miR-503-5p	1.34	0.42	0.5695
miR-140-5p	-1.19	-0.25	0.5788
RNU5G	-1.44	-0.52	0.6036
miR-328-3p	1.50	0.59	0.6064
miR-101a-3p	1.11	0.15	0.6323
miR-19b-3p	1.23	0.30	0.6357
miR-23a-3p	-1.07	-0.10	0.6381
miR-199a-3p	1.07	0.09	0.6516
miR-200b-3p	1.11	0.16	0.6662
miR-25-3p	1.14	0.18	0.6798
let-7a-1-3p	1.18	0.23	0.6823
miR-375-3p	-1.15	-0.20	0.6839
miR-331-3p	-1.22	-0.29	0.6871
miR-29b-3p	1.32	0.40	0.6919
miR-361-5p	1.08	0.11	0.6968
miR-203-3p	-1.07	-0.10	0.7237
miR-423-3p	-1.15	-0.20	0.7256
miR-30c-2-3p	-1.19	-0.25	0.7331
let-7b-5p	1.16	0.21	0.7345
miR-23b-3p	1.03	0.05	0.7354
miR-652-3p	-1.09	-0.12	0.7438
miR-322-5p	1.07	0.10	0.7556
miR-146a-5p	-1.09	-0.13	0.7614
miR-181a-5p	1.12	0.16	0.7757
miR-345-5p	1.12	0.16	0.7834
miR-17-5p	1.14	0.19	0.7853
miR-378a-3p	-1.04	-0.05	0.7872
miR-192-5p	-1.05	-0.07	0.7874
miR-30d-5p	-1.05	-0.07	0.7910
miR-320-3p	-1.08	-0.11	0.7968
miR-20a-5p	1.07	0.10	0.8074
miR-126a-3p	-1.07	-0.10	0.8078
miR-18a-5p	1.09	0.12	0.8180
miR-187-3p	1.12	0.16	0.8227
let-7i-5p	-1.06	-0.08	0.8318
miR-874-3p	1.11	0.15	0.8332
miR-26b-5p	-1.09	-0.12	0.8393
miR-130a-3p	-1.06	-0.09	0.8465
miR-151-3p	1.07	0.09	0.8753
miR-100-5p	1.02	0.03	0.8799
miR-201-5p	-1.06	-0.08	0.8811
miR-32-5p	-1.10	-0.14	0.8846
miR-340-5p	1.08	0.12	0.8872
miR-145a-5p	-1.03	-0.04	0.9184
miR-195a-5p	-1.02	-0.02	0.9353
miR-326-3p	1.03	0.04	0.9616

rno-miR-214-3p	1.02	0.03	0.9657
RNU1A1	-1.04	-0.05	0.9677
miR-150-5p	-1.01	-0.02	0.9726
miR-382-5p	1.01	0.02	0.9753
miR-1a-3p	-1.02	-0.02	0.9759
miR-484	1.02	0.03	0.9767
miR-338-3p	1.01	0.01	0.9814
miR-139-5p	1.01	0.01	0.9893
miR-532-5p	-1.00	0.00	1.0000

Table S12. Correlation coefficients between clinical parameters and selected circulating miRNAs in a human cohort. Boldface indicates p<0.05 (n=40). WHR= waist/hip ratio; FPI= fasting plasma insulin; FPG= fasting plasma glucose; G120= glucose at the 2h point during an oral glucose tolerance test.

	miR-122-5p	*miR-378-3p*	*miR-19b-3p*	*miR-101-3p*	*miR-107-3p*	*miR-223-3p*	*miR-22-3p*
**BMI**	-0.130	0.218	-0.059	0.002	-0.175	-0.202	**0.351**
**WHR**	0.065	0.301	0.087	-0.046	-0.184	0.002	**0.405**
**FLI**	0.170	0.230	-0.034	-0.111	-0.289	-0.127	**0.409**
**TG (mmol/L)**	**0.340**	0.090	0.032	-0.197	-0.217	-0.026	**0.322**
**CHOL (mmol/L)**	-0.082	**-0.368**	0.043	-0.009	0.113	-0.006	0.170
**LDL (mmol/L)**	-0.113	-0.248	0.036	0.118	0.027	-0.030	0.232
**HDL (mmol/L)**	-0.277	**-0.406**	-0.076	0.020	**0.403**	0.112	-0.285
**FPI (mIU/L)**	0.152	**0.397**	-0.209	0.107	-0.118	-0.301	**0.436**
**FPG (mg/dl)**	-0.058	0.057	0.099	-0.179	0.010	**-0.413**	**0.462**
**G120 (mg/dl)**	0.116	0.069	0.078	0.058	-0.177	-0.116	**0.403**
**HOMA-IR**	0.101	**0.356**	-0.171	0.008	-0.090	**-0.387**	**0.494**

Table S13. Main clinical features and *miR-22-3p* values of the study population.

	FLI<60 (n=13)	FLI>60 (n=27)	p
**AGE (yr)**	55.38 ± 1.60	52.75 ± 0.90	0.132
**FLI**	44.55 ± 3.66	83.58 ± 2.21	**0.000**
**BMI (kg/m^2)**	27.10 ± 0.61	31.05 ± 0.58	**0.000**
**Cholesterol (mmol/L)**	4.96 ± 0.27	5.04 ± 0.15	0.772
**HDL (mmol/L)**	1.17 ± 0.09	1.04 ± 0.05	0.164
**LDL (mmol/L)**	2.77 ± 0.19	2.77 ±0.11	0.993
**TG (mmol/L)**	1.31 ± 0.18	2.34 ± 0.26	**0.013**
**Insulin (mIU/L)**	11.35 ± 1.78	14.88 ± 1.41	0.161
**Glucose (mg/dL)**	95.53 ± 3.50	99.51 ± 2.91	0.423
**HOMA-IR**	2.72 ±0.45	3.75 ± 0.44	0.161
***miR-22-3p* (dCt)**	1.07 ± 0.08	1.38 ± 0.10	0.050

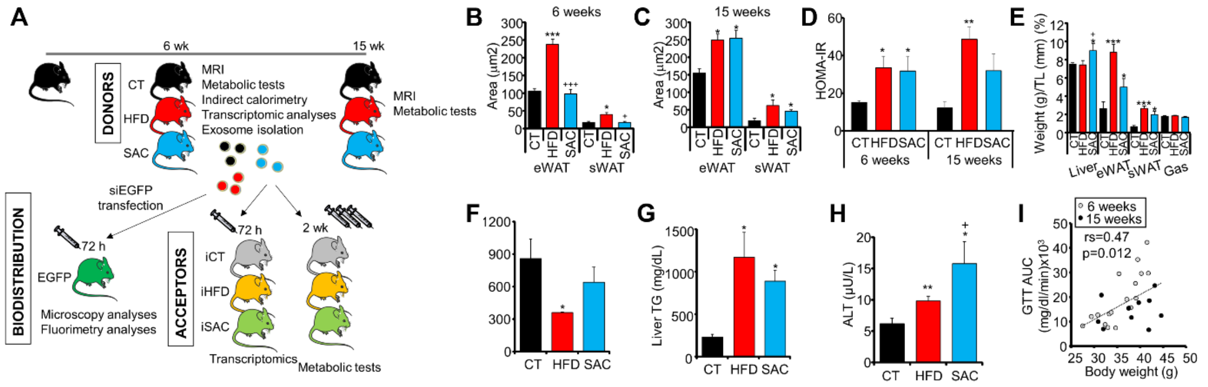

Figure S1. High-fat and high-sucrose diets lead to a convergent obese phenotype over time. (**A**) Scheme of animal experiments. (**B, C**) Quantification of fat distribution of mice fed either standard chow (CT), a high-fat diet (HFD) or standard chow with drinking water supplemented with 50% sucrose (SAC) after 6 (**B**) and 15 weeks (**C**) of diet, according to the MRI scans shown in Figure 1E. (**D**) HOMA-IR of the same mice as calculated from the values of glucose and insulin shown in Figure 1. (**E**) Percentage of tissue weight relative to tibia length at the time of sacrifice of CT, HFD and SAC mice after 6 weeks. (**F**) Adipocyte number in the eWAT of the same mice as determined from the IHC shown in figure 1O. (**G, H**) Liver TG content (**G**) and plasma ALT activity of the same mice (**H**). (**I**) Correlation of body weight with the glucose AUC of the GTTs performed at 6 weeks (white circles) and 15 weeks (black circles). n=3/group (B, C, F), n=6/group (D, E, G, H), n=12/group (6 weeks) and n=3/group (15 weeks) (I). *p<0.05, **p<0.01, ***p<0.005 with respect to CT group. $^{+}$p<0.05, $^{++}$p<0.01, $^{+++}$p<0.005 with respect to HFD group.

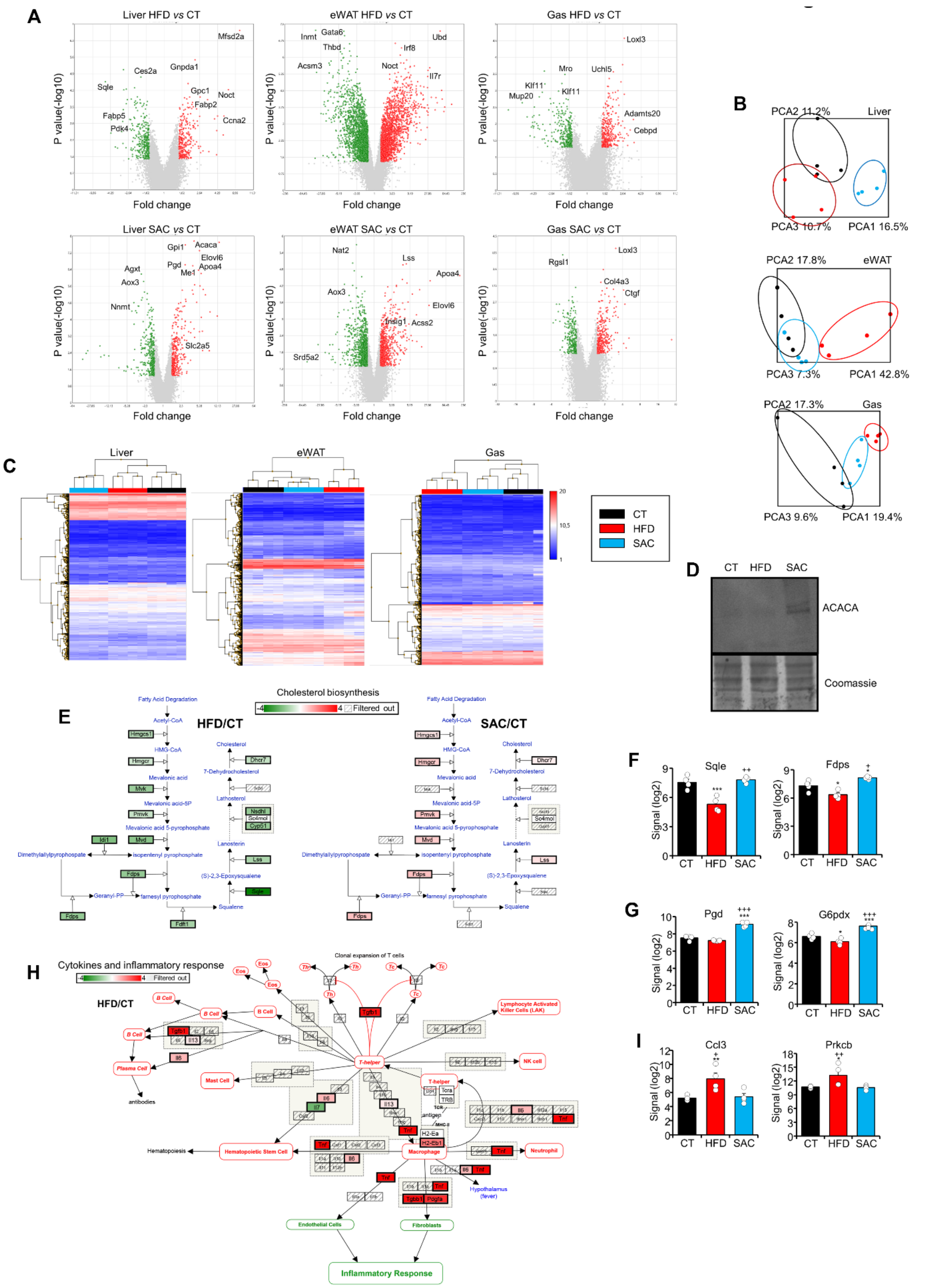

Figure S2. HFD and SAC induce distinct gene expression profiles in eWAT and liver. (**A**) Volcano plots of HFD/CT (upper panels) and SAC/CT (lower panels) liver, eWAT and gastrocnemius muscle. (**B**) PCA of liver (upper panel), eWAT (middle panel) and muscle (lower panel). (**C**) Heatmaps showing hierarchical clustering of differentially expressed genes comparing CT, HFD and SAC groups. (**D**) Key DNL regulator ACACA is increased in SAC liver. (**E, F**) The cholesterol biosynthesis pathway is

decreased in HFD liver and increased in SAC liver. (**G**) Genes of the pentose phosphate pathway are increased in liver of SAC mice. (**H, I**) The cytokine and inflammatory response pathway is increased in HFD eWAT. n=4/group (A-I). *p<0.05, **p<0.01, ***p<0.005 with respect to CT group. $^{+}$p<0.05, $^{++}$p<0.01, $^{+++}$p<0.005 with respect to HFD group.

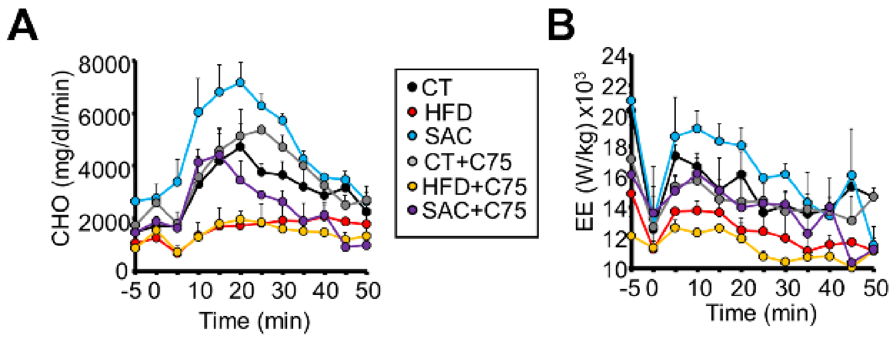

Figure S3. SAC mice rely on DNL to maintain glucose homeostasis. (**A, B**) Oral administration of glucose increases carbohydrate oxidation (**A**) and energy expenditure (**B**) in SAC mice to levels above CT mice, as calculated by VO_2 and VCO_2 values measured by indirect calorimetry, and FASN inhibitor C75 pretreatment blunts the increase. n=3/group.

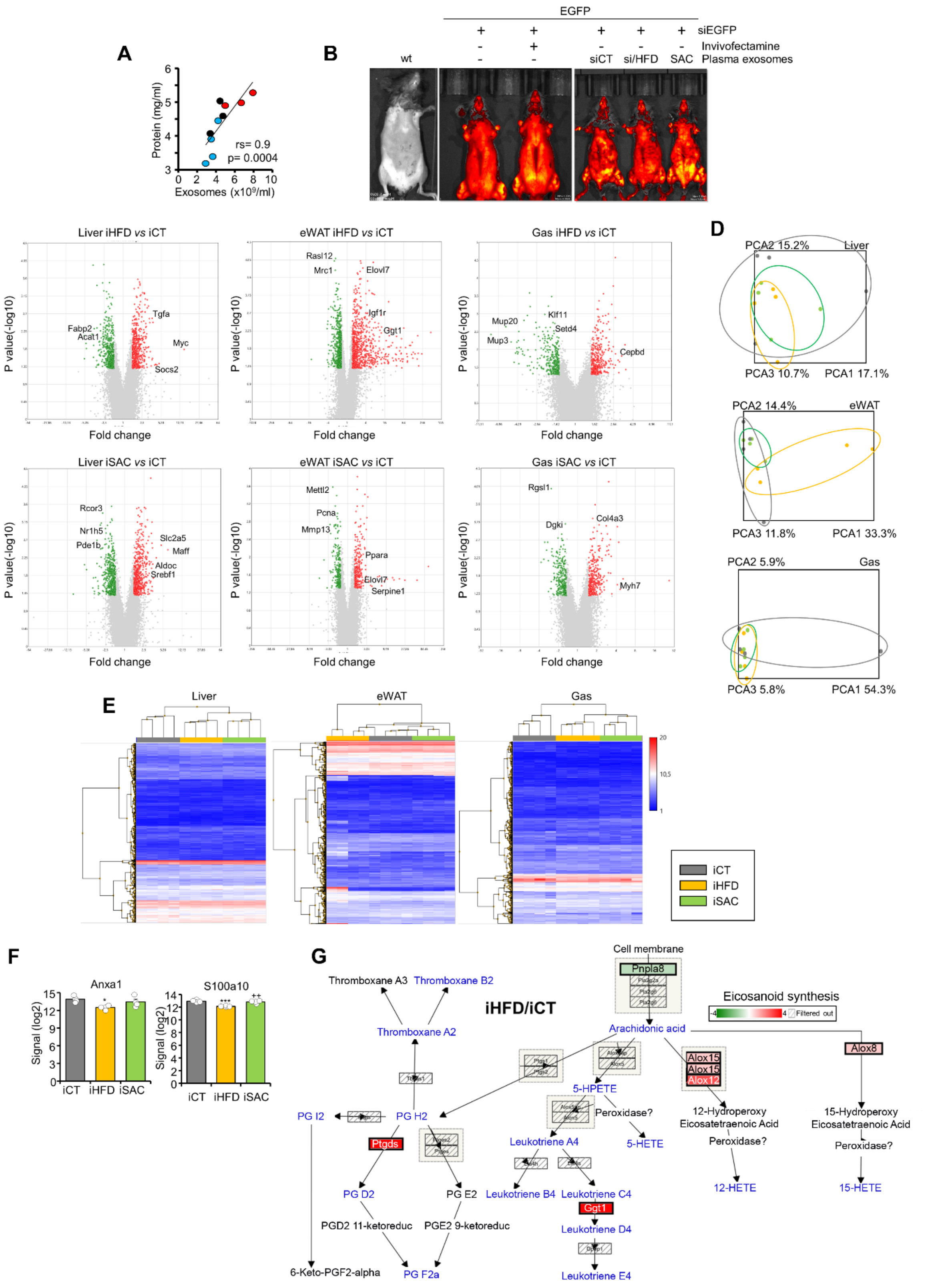

Figure S4. Injection of HFD and SAC plasma exosomes reproduce transcriptomic changes observed in donor mice. (**A**) Exosome number quantified by Exocet ELISA significantly correlates with total exosome protein as determined by Bradford assay. (**B**) Transgenic mice expressing green fluorescent protein were injected with exosomes isolated from the plasma of CT, HFD and SAC mice and transfected with siEGFP. The same siRNA coupled to Invivofectamine was used as positive control.

Global distribution of fluorescence was determined by IVISS scanning. (**C**) Volcano plots of liver, eWAT and gastrocnemius muscle of mice injected with HFD plasma exosomes (iHFD) (upper panels) or SAC exosomes (iSAC) (lower panels) as compared to those injected with CT exosomes (iCT). (**D**) PCA of liver (upper panel), eWAT (middle panel) and muscle (lower panel). (**E**) Heatmaps showing hierarchical clustering of differentially expressed genes comparing iCT, iHFD and iSAC groups. (**F, G**) Prostaglandin genes are decreased (**F**) and eicosanoid synthesis is increased in eWAT of iHFD mice (**G**). n=3/group (A, B), n=4/group (C-G). *p<0.05, **p<0.01, ***p<0.005 with respect to CT group. $^{+}$p<0.05, $^{++}$p<0.01, $^{+++}$p<0.005 with respect to HFD group.

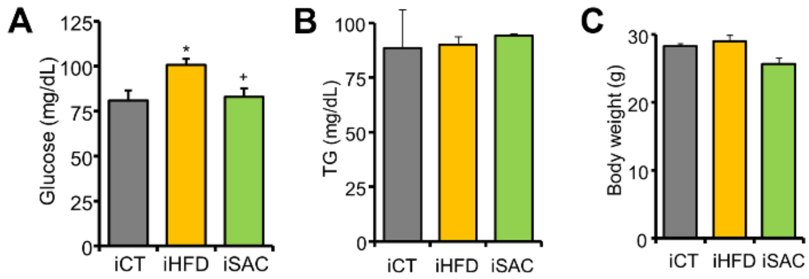

Figure S5. Injection of HFD and SAC plasma exosomes reproduce the metabolic phenotype observed in donor mice. (**A-C**) Chronic treatment of control mice with exosomes isolated from plasma of CT, HFD and SAC mice show increased fasting plasma glucose in iHFD mice (**A**) with no changes in circulating triglyceride (**B**) or body weight (**C**) for any group. n=4/group. *p<0.05 with respect to iCT group. +p<0.05 with respect to iHFD group.

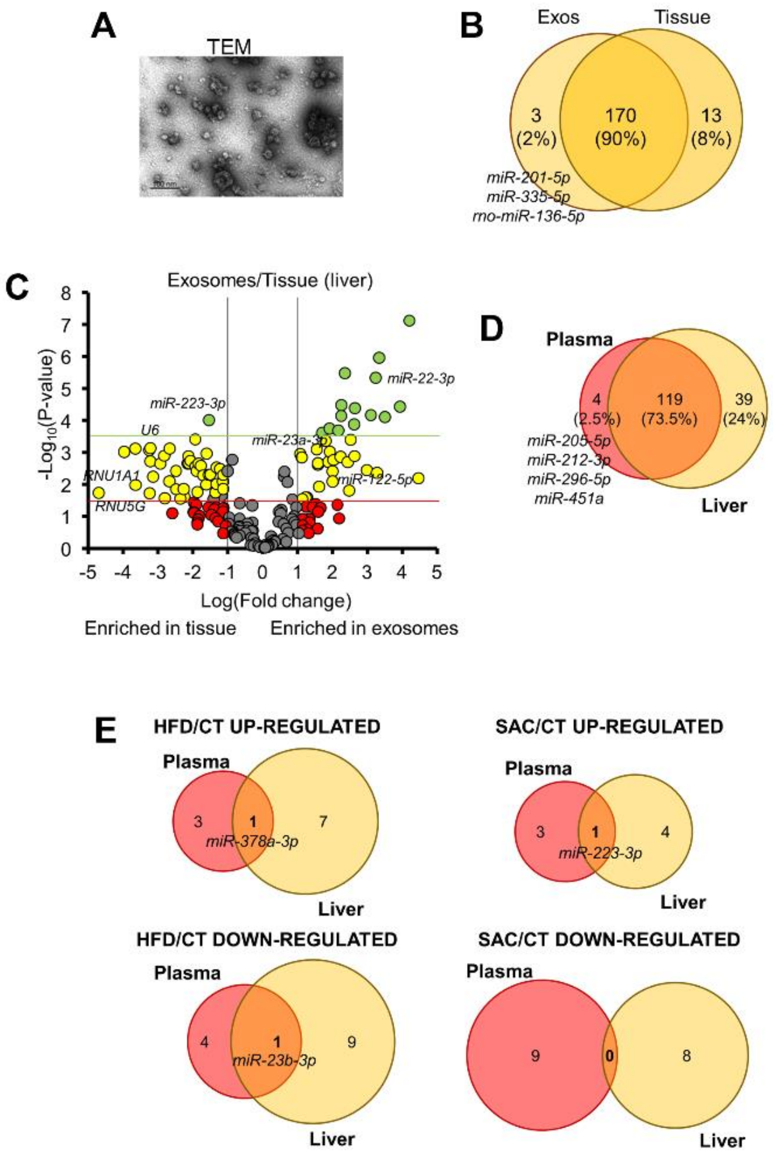

Figure S6. The miRNA profile differs between HFD and SAC plasma and liver exosomes. (**A**) Liver exosomes are characterized by transmission electron microscopy. (**B**) Comparison of the detected miRNAs in control liver exosomes and tissue. (**C**) Volcano plot of liver exosomes and liver tissue shows enrichment of snRNAs in tissue as compared to exosome samples. (**D**) Comparison of the miRNAs detected by RT-PCR in plasma and tissue exosomes of CT mice. (**E**) Comparison of the upregulated and downregulated miRNAs in plasma and liver exosomes of HFD (left panels) and SAC mice (right panels). n=4/group (A-E).

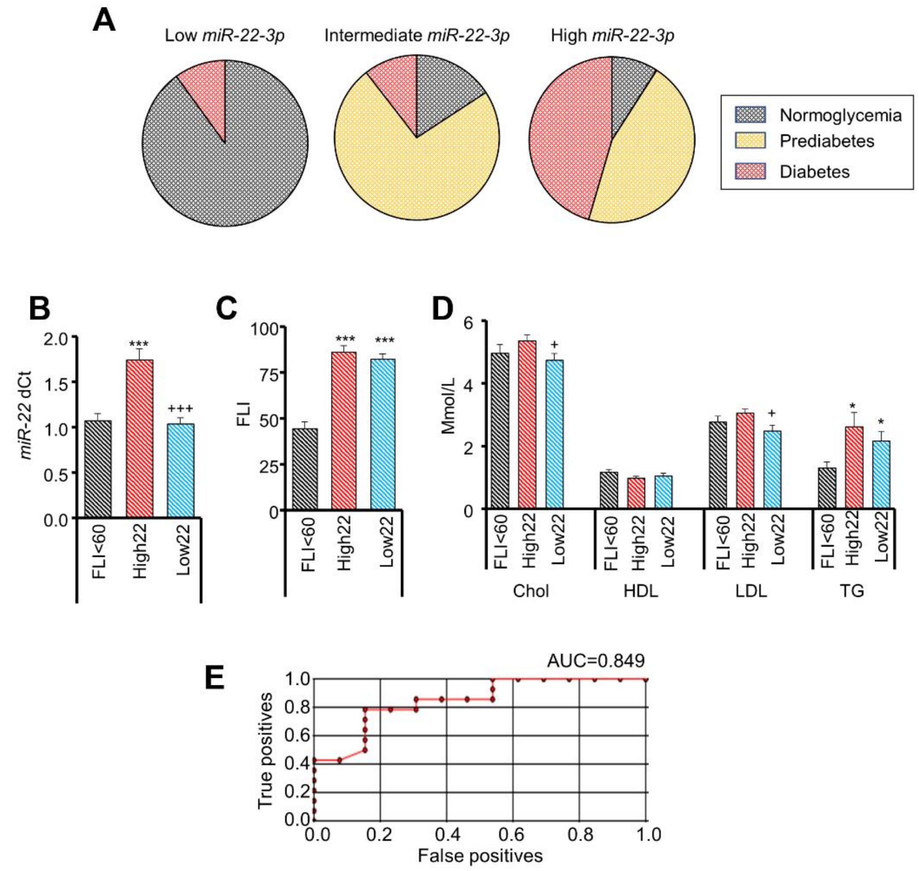

Figure S7. Circulating *miR-22-3p* can be used as a biomarker for MAFLD stratification. (**A**) Subjects with increasing levels of circulating *miR-22-3p* (low < 1.0 < intermediate < 1.4 < high, relative values) display a more unfavorable clinical diagnosis. (**B**) Subjects with a low fatty liver index (FLI<60) show low levels of circulating *miR-22-3p*, whereas subjects with FLI>60 can be separated into two subgroups with either high (High22) or low (Low22) circulating *miR-22-3p* levels. (**C, D**) Individuals in the High22 group show equivalent FLI (**C**) but a worse lipid profile (**D**) than those in the Low22 group. (**E**) ROC analysis of circulating *miR-22-3p* as a parameter to discern HOMA-IR>2.7 in patients with FLI>60. n=40 (A-D), n=27 (E). *p<0.05, ***p<0.005 with respect to FLI<60 group. +p<0.05, +++p<0.005, with respect to High22 group.
